# Elimination of charge-carrier trapping by molecular design

**DOI:** 10.1038/s41563-023-01592-3

**Published:** 2023-06-29

**Authors:** Oskar Sachnik, Xiao Tan, Dehai Dou, Constantin Haese, Naomi Kinaret, Kun-Han Lin, Denis Andrienko, Martin Baumgarten, Robert Graf, Gert-Jan A. H. Wetzelaer, Jasper J. Michels, Paul W. M. Blom

**Affiliations:** Max Planck Institute for Polymer Research, Mainz, Germany

**Keywords:** Electronic devices, Organic molecules in materials science

## Abstract

A common obstacle of many organic semiconductors is that they show highly unipolar charge transport. This unipolarity is caused by trapping of either electrons or holes by extrinsic impurities, such as water or oxygen. For devices that benefit from balanced transport, such as organic light-emitting diodes, organic solar cells and organic ambipolar transistors, the energy levels of the organic semiconductors are ideally situated within an energetic window with a width of 2.5 eV where charge trapping is strongly suppressed. However, for semiconductors with a band gap larger than this window, as used in blue-emitting organic light-emitting diodes, the removal or disabling of charge traps poses a longstanding challenge. Here we demonstrate a molecular strategy where the highest occupied molecular orbital and lowest unoccupied molecular orbital are spatially separated on different parts of the molecules. By tuning their stacking by modification of the chemical structure, the lowest unoccupied molecular orbitals can be spatially protected from impurities that cause electron trapping, increasing the electron current by orders of magnitude. In this way, the trap-free window can be substantially broadened, opening a path towards large band gap organic semiconductors with balanced and trap-free transport.

## Abstract (web summary)

Extrinsic impurities may trap electrons or holes leading to imbalanced charge transport in wide band gap organic semiconductors. Here the authors propose a bottom-up design strategy by spatially separating HOMO and LUMO orbitals to avoid charge trapping, enhancing charge transport by orders of magnitude.

## Main

Organic semiconductors often show relatively poor charge transport properties compared with their inorganic counterparts. There are two fundamental reasons limiting their charge transport: the first one is a low carrier mobility, arising from the fact that organic molecules are held together by weak van der Waals and π–π non-covalent forces, making them susceptible to energetic and structural disorder. As a result, the charge transport is governed by hopping between localized states, which is less efficient than band conduction in crystalline inorganic semiconductors^1^. In the past three decades, by optimizing the molecular packing^2^, mobility values exceeding 10 cm^2^ V^−1^ s^−1^ for both *n*- and *p*-type organic semiconductors have been reported^3^. A second reason leading to poor charge transport, even for high mobility materials, is trapping of charge carriers by impurities. In this case, only a small fraction of the injected carriers contribute to the charge transport. Trapping of either electrons or holes is the main cause of imbalanced transport in organic semiconductors^4,5^. Recently, an energy window was identified, inside which organic semiconductors are not susceptible to charge trapping. Trap-free bipolar charge transport can be accomplished when the electron affinity (EA) of the organic semiconductor is higher than 3.5 eV and the ionization energy (IE) is lower than 6.0 eV^6^. This universal window, which applies to semiconducting polymers as well as to small molecules, indicates that the extrinsic charge traps in organic semiconductors share a common origin. Electron trapping has been attributed to oxygen-related impurities^7^, whereas hole traps are linked to water clusters^6,8^. Furthermore, next to oxygen, also omnipresent water has been proposed as a possible source for electron trapping^9^. However, the relation between processing conditions and trapping is still under debate.

The fundamental question remains whether it is possible to achieve intrinsic trap-free transport of both electrons and holes for organic semiconductors with a band gap larger than the trap-free window of 2.5 eV. In that case, either the highest occupied molecular orbital (HOMO) or lowest unoccupied molecular orbital (LUMO) or both are outside the trap-free window. As trapping is detrimental to the efficiency of single-layer organic light-emitting diodes (OLEDs)^10^, trap-free ambipolar charge transport is a prerequisite to achieve highly efficient devices^11–13^. The limited width of 2.5 eV of the energy window implies that for large band gap materials, as used in blue OLEDs, it is fundamentally not possible to obtain trap-free transport of both carriers, thus preventing the realization of efficient printed single-layer blue OLEDs in future. In addition, for multilayer blue OLEDs, the imbalance in electron and hole transport in the large band gap host^14^ leads to an unevenly distributed emission zone as well as to unwanted interactions of excess holes with excitons, which decrease the operational lifetime of the device^15,16^.

Here we demonstrate an approach on how simultaneous trap-free electron and hole transport can be intrinsically accomplished in wide band gap organic semiconductors through molecular design. The basic idea is to use donor–acceptor based molecules, with the LUMO localized on the acceptor part and the HOMO localized on the donor part. By shielding the acceptor core where the electron transport takes place with the donor moieties, the interaction of impurities with the LUMO leading to electron trapping can be effectively blocked. This work therefore represents a universal molecular bottom-up concept to eliminate the detrimental effects of external impurities in organic semiconductors.

### Materials

**Fig. 1:**
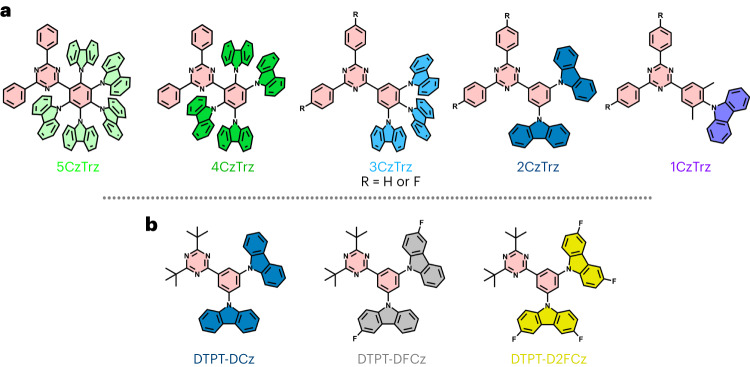
Molecular structures. **a**,**b**, Structural formulas of series 1 consisting of 1CzTrz, 2CzTrz, 3CzTrz, 4CzTrz and 5CzTrz (**a**), and series 2 consisting of DTPT-DCz, DTPT-DFCz and DTPT-D2FCz (**b**). The triazine acceptor is indicated in pink, whereas the colour of the carbazole donor is varied, corresponding to the symbols of the *J–V* characteristics in Fig. 2.

The basic structure of a series of blue-emitting molecules presented in this study consists of a triazine (Trz) acceptor linked to carbazole (Cz) donor(s) by a phenylene linker (Fig. 1). Triazine-based materials are well known for their efficient transport of electrons^17^. A similar combination of triazine and carbazole has been used as blue emitter exploiting thermally activated delayed fluorescence, where it was shown that an increase of the amount of Cz donor units from two to three led to an enhancement of the OLED efficiency^18^. However, the individual charge transport properties of these CzTrz-based materials were not addressed.

Two series of organic semiconducting blue emitters have been synthesized (Fig. 1) and were investigated in terms of electron transport and molecular arrangement in thin films. In the first series, the blue emitters share the same triazine acceptor but a different number of donating carbazole units (bridged by a phenylene linker): 9-(4-(4,6-diphenyl-1,3,5-triazin-2-yl)−2,6-dimethylphenyl)-9*H*-carbazole (1CzTrz), 9,9′-(5-(4,6-diphenyl-1,3,5-triazin-2-yl)-1,3-phenylene)bis(9*H*-carbazole) (2CzTrz), 9,9′,9″-(5-(4,6-diphenyl-1,3,5-triazin-2-yl)benzene-1,2,3-triyl)tris(9*H*-carbazole) (3CzTrz), 9,9′,9″,9‴-(3-(4,6-diphenyl-1,3,5-triazin-2-yl)benzene-1,2,4,5-tetrayl)tetrakis(9*H*-carbazole) (4CzTrz) and 9,9′,9″,9‴,9″″-(6-(4,6-diphenyl-1,3,5-triazin-2-yl)benzene-1,2,3,4,5-pentayl)pentakis(9*H*-carbazole) (5CzTrz). The second series consists of the same triazine acceptor and two donating carbazole units, also bridged by a phenylene linker, but with different number of fluorine substituents on the carbazole unit: 9,9′-(5-(4,6-di-tert-butyl-1,3,5-triazin-2-yl)-1,3-phenylene)bis(9H-carbazole) (DTPT-DCz), 9,9′-(5-(4,6-di-tert-butyl-1,3,5-triazin-2-yl)-1,3-phenylene)bis(3-fluoro-9H-carbazole) (DTPT-DFCz) and 9,9′-(5-(4,6-di-tert-butyl-1,3,5-triazin-2-yl)-1,3-phenylene)bis(3,6-difluoro-9H-carbazole (DTPT-D2FCz).

First, we measured the IE and EA of the five 1–5CzTrz compounds (Fig. 1a) using a combination of ultraviolet photoelectron spectroscopy (UPS) (Supplementary Fig. 1 and Supplementary Table 1), cyclic-voltammetry measurements (Supplementary Fig. 2) and solution ultraviolet–visible absorption and photoluminescence (PL) measurements (Supplementary Fig. 3). The IE of this series of molecules of ~5.8 eV is within the trap-free energy window, meaning that trap-free hole transport is expected for all of these compounds^6^. The measured EA (Supplementary Table 1) amounts to 3.1 ± 0.1 eV for all compounds, clearly well below the value of 3.6 eV for trap-free electron transport^6^. Hence, in contrast to the hole current, the electron current is expected to be strongly trap-limited in all cases based on energy-level considerations.

### Electron transport

**Fig. 2:**
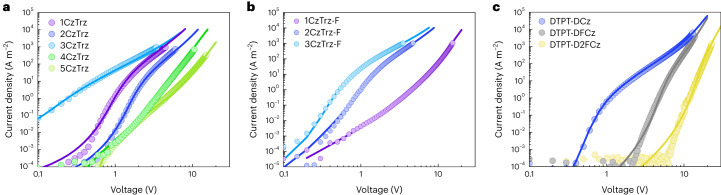
Electron current of CzTrz-based and DTPT-DFCz-based compounds. **a**, Experimental (symbols) and simulated (lines) current density (*J)*–voltage (*V*) characteristics of 1CzTrz (94 nm), 2CzTrz (100 nm), 3CzTrz (98 nm), 4CzTrz (79 nm) and 5CzTrz (102 nm). **b**, Experimental (symbols) and simulated (lines) *J*–*V* characteristics of the fluorinated compounds 3CzTrz-F (118 nm), 2CzTrz-F (91 nm) and 1CzTrz-F (95 nm). **c**, Experimental (symbols) and simulated (lines) *J*–*V* characteristics of DTPT-DCz (106 nm), DTPT-DFCz (95 nm) and DTPT-D2FCz (99 nm).

Subsequently, we investigated the electron transport in the emitters 1CzTrz–5CzTrz using electron-only devices. We refer to the Methods for details. In Fig. 2a, the measured (symbols) electron current density ($J$) as a function of voltage ($V$) for the 1–5CzTrz series is displayed. The thickness of the investigated devices is in the range of 80–100 nm. Despite the fact that all molecules comprise the same donor and acceptor moieties, we observe a four to five orders of magnitude difference in the electron current density, depending on the number of donor substituents. Intriguingly, the electron current in 3CzTrz shows a quadratic dependence of the current on voltage, indicative of trap-free space-charge-limited electron transport, despite having its LUMO energy outside the trap-free window. The fact that the lower current density for the other compounds is accompanied by an increased voltage dependence of the current indicates that the strong reduction in transport is caused by electron trapping^4^.

Similar behaviour occurs for the fluorinated 1–3CzTrz-F series: the EAs as obtained from cyclic-voltammetry measurements (Supplementary Table 1) range from 2.7 eV to 2.9 eV and are all far outside the trap-free window. However, as shown in Fig. 2b, also for this series the compound with three Cz units 3CzTrz-F shows nearly trap-free electron transport, whereas the electron current of 1CzTrz-F is strongly reduced, showing a steep *J–V* curve with a slope of around 6 in the log(*J*)–log(*V*) plot. For both series an optimum electron current is reached for three Cz units, for which a nearly trap-free space-charge limited current ($J \sim {V}^{2}$) is measured. A further decrease or increase in the number of Cz moieties results in more severe electron trapping. To quantify the trap density, the *J–V* characteristics are modelled (lines, Fig. 2) with a previously developed drift-diffusion model^19^. The electron mobility is obtained from the quadratic trap-free regime observed for the 3CzTrz molecule and amounts to 2 × 10^−9^ m^2^ V^−1^ s^−1^. The currents of the other compounds are then described by the addition of electron traps, assuming a Gaussian energy distribution of trap states^4^. The trap concentrations and transport parameters for the 1–5CzTrz compounds and 1–3CzTrz-F series are given in Supplementary Tables 2–4, respectively. By studying the dependence on layer thickness and temperature, we confirmed that the observed difference of orders of magnitude in the electron current is not the result of a variation in injection barrier or built-in voltage *V*_bi_ (Supplementary Figs 4–7). Ohmic contacts have been realized by using a 1,3,5-Tris(1-phenyl-1*H-*benzimidazol-2-yl)benzene (TPBi) tunnel barrier to decouple the semiconductor from the electrode^20^.

### Energy distribution

To obtain more insight into the molecular mechanism of trapping, we have as the next step computed the density of states (DOS) of the five 1–5CzTrz and three 1–3CzTrz-F compounds assuming the films to be amorphous (disordered molecular arrangement), with traps due to molecular oxygen (Supplementary Table 6; for further computational details, see Supplementary Information). The calculated ionization energies (Supplementary Table 7) agree well with the experimentally obtained numbers (Supplementary Table 1). The DOS distribution of the EA of molecular oxygen and amorphous 1–5CzTrz (LUMO) show that, as expected, the value of the EA increases with increasing number of carbazoles from 1CzTrz to 3CzTrz (Supplementary Fig. 8). As the EA distributions of O_2_ are not notably different (Supplementary Table 8), the increased EA results in reduced trapping. Further increase of the number of carbazoles (4–5CzTrz) results in lower EA and enhanced trapping (Supplementary Table 7), which agrees with the trend of trap densities obtained from the drift-diffusion model (Supplementary Table 2). In contrast, for the fluorinated 1–3CzTrz-F compounds, the value of EA and distributions of O_2_ do not notably vary, such that identical trapping is expected for all compounds (Supplementary Tables 9 and 10). This is clearly in contrast with the strong variation in electron current, shown in Fig. 2b. This suggests that it is not only the energetics of the molecules being responsible for the observed large variation in electron transport of the various CzTrz-based compounds. An open question is whether extrinsic electron trapping is also strongly dependent on the molecular arrangement of the molecules in the solid film. Of course, this can only be the case if the films are not fully amorphous but rather show molecular ordering to some extent, for instance in a coexisting phase comprising (nano-)crystalline domains^21,22^.

### Morphology

To investigate the molecular ordering in the compounds with three or fewer carbazole units, we subjected the fluorinated monocarbazole and tricarbazole species to a comparative analysis using two complementary experimental methods, namely, X-ray diffraction (XRD) and magic angle spinning (MAS) solid-state nuclear magnetic resonance (SS-NMR)^23,24^. XRD was applied to single crystals, grown by anti-solvent diffusion as described in Methods. The SS-NMR analysis was used to establish the extent of ordering in the evaporated thin film. The reason to select the 1CzTrz-F and 3CzTrz-F for this analysis is that the fluorine substituent provides for a highly sensitive marker, owing to the fact that its natural isotope (^19^F) has spin of $+1/2$^25^. Furthermore, the 1CzTrz-F and 3CzTrz-F show a few orders of magnitude difference in their electron transport, with the transport in 1CzTrz-F heavily trap limited and a nearly trap-free transport in 3CzTrz-F. In addition, we managed to grow crystals of sufficient quality for XRD of both 1CzTrz-F and 3CzTrz-F compounds.

**Fig. 3:**
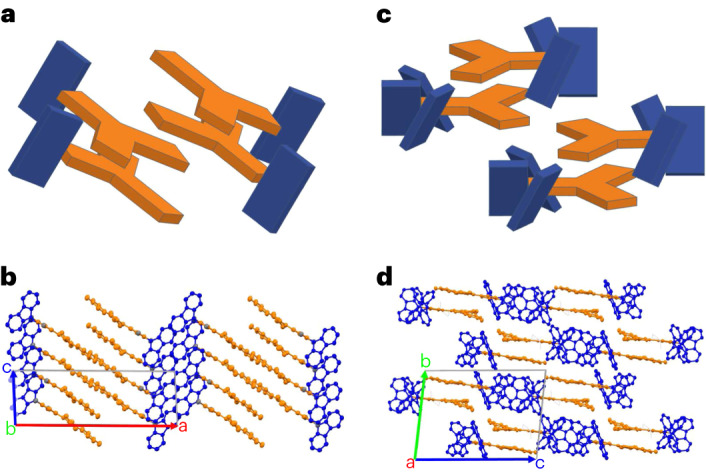
Molecular structures obtained from XRD. **a**–**d**, Crystal structures of the 1CzTrz-F (**a**,**b**) and 3CzTrz-F (**c**,**d**) compounds, determined by XRD. **a**,**c**, Diagrams of the two dimers of both crystallographic unit cells to show the molecular packing. **b**,**d**, Spatial arrangement of the acceptor–donor contacts in the 3D crystal structure. The triazine acceptor and the carbazole donor units are coloured orange and blue, respectively. The green features in **d** indicate co-crystallized chloroform molecules.

To characterize the structure of the single crystals, XRD analysis (see for details Supplementary Table 15) revealed the space group for 1CzTrz-F to be P1 (monoclinic), with no co-crystallized solvent molecules. As displayed in Fig. 3a,b, the unit cell contains four molecules, paired into two dimers with antiparallel stacking of the triazine planes and considerable spatial overlap between the outer phenyl groups. The distance between the molecular planes is 3.5 Å. The carbazole units are arranged in an angle close to 90° relative to the connecting phenyl ring, caused by the steric hindrance of the two ortho-methyl groups.

Furthermore, as shown in Fig. 3a, the Cz units of the species within one dimer are rotated relative to each other by approximately 60^o^ around the centres of the triazine rings. The three-dimensional arrangement leads to a structure with alternating two-dimensional layers of carbazole and triazine rings perpendicular to the crystallographic *a* axis. Dimer formation agrees with earlier work by Monkman et al.^26^ who demonstrated that dimers are responsible for the spectral shifts observed in carbazole-based thermally activated delayed fluorescence emitters^26^. The 3CzTrz-F crystal structure falls in the P2_1_/c space group (triclinic), showing solvent co-crystallization. Again, we encounter four molecules in a single unit cell (Fig. 3c,d), showing a dimeric arrangement. However, now the dimers are formed by antiparallel molecular alignment, and the stacking involves not only the triazine rings but also the outer phenyl rings. The torsion angles of the carbazole groups are 120 ± 10°, possibly resulting from weak π–π interaction of neighbouring units bound to the same phenyl ring. The stacking of neighbouring molecules is slightly tilted, and the π systems of adjacent molecules do not perfectly superimpose. The stacking of neighbouring molecular planes, however, connects molecules within a plane, forming a one-dimensional double layer of acceptor units along the crystallographic *a* axis. The distance between the molecular planes is 3.4 Å. In summary, 3CzTrz-F shows an inclined face-to-face stacking of the phenyl substituted triazine cores along the crystallographic *a* axis, which effectively may act as a ‘tunnel’ for electron transport, crowded by carbazole units. In contrast, in 1CzTrz-F such crowding is lacking. We therefore propose that the origin of the difference in the electron trapping between the monocarbazole and tricarbazole species is the result of the stacking geometry: ‘open’ for the monocarbazole species and ‘closed’ for the tricarbazole compounds, meaning that in the latter the electron transporting core is effectively shielded from interactions with extrinsic contaminants such as oxygen.

In what follows, we confirm using MAS SS-NMR that molecular ordering indeed occurs in vapor-deposited material, supporting our explanation of differences in electron transport in terms of differences in molecular packing. The spectra of 1CzTrz-F (^1^H and ^19^F) and 3CzTrz-F (^1^H and ^19^F) are plotted in Supplementary Fig. 15. For 1CzTrz-F the ^19^F signal is split despite the symmetry in the molecular structure (Supplementary Fig. 15a). This shows that there is a preferred local molecular packing arrangement that breaks the molecular symmetry of the two ^19^F sites in the molecule. In a random or fully amorphous arrangement, there should also be molecules where the molecular symmetry is preserved, and then only a single very broad peak should be seen. Similar as for 1CzTrz also in the ^19^F MAS NMR spectrum of the 3CzTrz-F (Supplementary Fig. 15b) compounds, two signals are observed, again pointing to a difference in the local chemical environment between the two ^19^F sites.

**Fig. 4:**
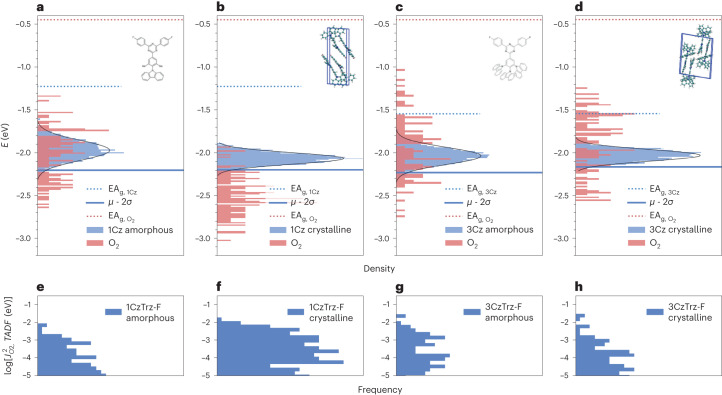
Calculated density-of-states distributions. **a**–**d**, The DOS of EA of amorphous 1CzTrz-F (**a**), crystalline 1CzTrz-F (**b**), amorphous 3CzTrz-F (**c**) and crystalline 3CzTrz-F (**d**). **e**–**h**, Magnitude of electronic transfer integral versus occurrence frequency in amorphous 1CzTrz-F (**e**), crystalline 1CzTrz-F (**f**), amorphous 3CzTrz-F (**g**) and crystalline 3CzTrz-F (**h**). The dotted lines are the gas-phases EA for molecular oxygen (EA_g__, O2_) and organic materials (EA_g__, 1Cz_). The blue solid line represents the energy *µ*_e_ − 2*σ*_e_, with *µ*_e_ corresponding to the average of the calculated solid-state EA values and *σ*_e_ the standard deviation of the Gaussian distribution (Table S9). The energy *µ*_e_ − 2*σ*_e_ is expected to correspond to the onset of the solid-state EA from UPS measurements.

Having established that there is a local ordering in the films, it is evident that the theoretical interpretation based on the amorphous phase of 1–5CzTrz (Supplementary Fig. 8) and 1–3CzTrz-F (Supplementary Fig. 9) should be handled with care, as the impact of molecular packing is not captured by these simulations. For this purpose, we also simulated the DOS of the crystalline phases using the structural data (Fig. 3) obtained from XRD for 1CzTrz-F and 3CzTrz-F (Supplementary Figs 10–13 and Supplementary Tables 11–13). As shown in Fig. 4, the EA distributions of oxygen are quite similar in crystalline and amorphous 3CzTrz-F. In contrast, for 1CzTrz-F the oxygen EAs are much higher in the crystalline phase than in the amorphous phase, implying deeper traps. The energetic shift of the EA distribution in the crystalline state (Supplementary Table 11) is largely attributed to a change in the electrostatic contribution to the EA, as seen in Supplementary Figs 10 and 11. Considering the fact that there are regions in organic thin films with molecular packing resembling the crystalline state, the deep O_2_ traps (Supplementary Table 12) in crystalline 1CzTrz-F result in a higher overall trap density compared with 3CzTrz-F, which agrees with the trend of trap densities obtained from the drift-diffusion model (Supplementary Table 4).

However, as shown in Fig. 4c,d energetic considerations alone cannot account for the trap-free transport observed in 3CzTrz-F. This clearly suggests that the molecular packing is also an essential ingredient to obtain a trap-free current due to shielding of the electron transporting core from impurities by the stacking geometry. To further elucidate the effectiveness of the O_2_ traps in the 1CzTrz-F and 3CzTrz compounds, we have evaluated the electronic transfer integrals representing the coupling between close-lying oxygen and CzTrz-F pairs (Fig. 4e–h). It is shown that the total coupling strength, represented by the area under the histogram, is largest in the 1CzTrz-F crystalline phase, thereby stabilizing the oxygen and resulting in oxygen becoming a deeper and more effective trap in the crystalline 1CzTrz-F.

### Effect of fluorine

To demonstrate the generality of our approach, we have applied the same strategy for obtaining trap-free transport in large energy gap organic semiconductors to a second series of blue-emitting materials, consisting of a triazine acceptor with two carbazole units with(out) fluorine constituents, DTPT-DCz, DTPT-DFCz and DTPT-D2FCz (Fig. 1b). It is expected that the addition of electronegative fluorine moieties will enhance both the ionization energy and EA of the molecules. This is indeed observed experimentally from cyclic-voltammetry measurements (Supplementary Table 5), where the EA is enhanced from 2.6 eV (DTPT-DCz, no fluorine) to 2.8 eV (DTPT-D2FCz, 4 fluorine). For similar oxygen levels, the strongest electron trapping is therefore expected for the non-fluorinated DTPT-DCz compound. However, experimentally, the opposite behaviour is observed, as shown in Fig. 2c. DTPT-DCz shows nearly trap-free transport, whereas with increasing number of fluorine constituents the electron current is strongly reduced. The reduced current of the fluorinated compounds is well described by drift-diffusion simulations combining Ohmic contacts and trapping; the resulting transport and trapping parameters are given in Supplementary Table 14.

To obtain further insight, we simulated the DOS of both the amorphous and crystalline phases of these three compounds, shown in Supplementary Fig. 14. According to the amorphous phase simulations, similar trapping behaviour would be expected for all compounds, clearly in disagreement with the experiment. Similar to the 1CzTrz-F compound (Fig. 4b) the EA distribution of oxygen is considerably lowered in the crystalline phase of DTPT-D2FCz (Supplementary Fig. 14f), leading to enhanced trapping. Thus, also for this compound the enhanced trapping in the crystalline phase is a result of a change in the energetics. However, similar to the 3CzTrz-F case, the occurrence of a trap-free current in DTPT-DCz cannot be explained by the simulations (Supplementary Fig. 14a,b). In both the amorphous and crystalline phase severe trapping is predicted. This again strongly suggests that another packing-related mechanism plays an important role.

**Fig. 5:**
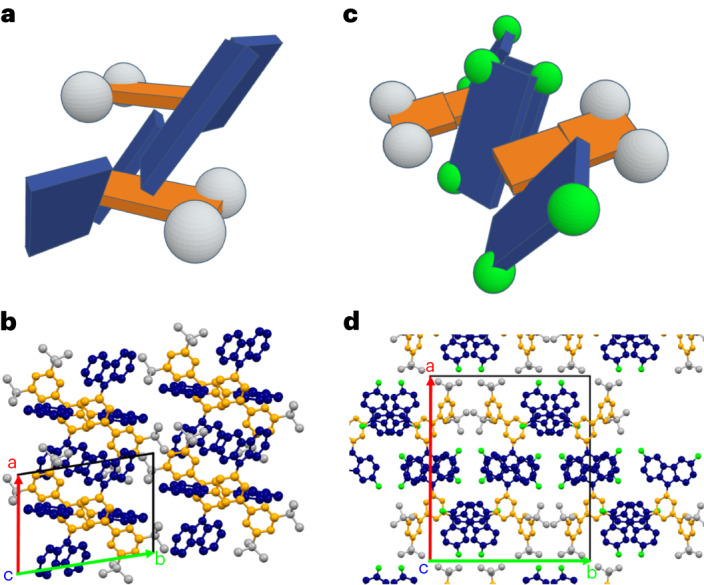
Molecular structures obtained from XRD. **a**–**d**, Crystal structures of the DTPT-DCz (**a**, **b**) and DTPT-D2FCz (**c**, **d**) compounds, determined by XRD. **a**,**c**, Diagrams of the two dimers of both crystallographic unit cells to show the molecular packing. **b**,**d**, Spatial arrangement of the acceptor–donor contacts in the 3D crystal structure. The triazine acceptor and the carbazole donor units are coloured orange and blue, respectively. The white features indicate the tert-butyl groups, whereas the green features in **d** indicate the fluorine atoms.

For this purpose, we have investigated the crystal structure using XRD on crystals of the tetrafluorinated DTPT-D2FCz and the non-fluorinated DTPT-DCz. In the latter sample, shown in Fig. 5a,b, the Cz substituents are arranged edge-on with a tilting angle of ~40° on top of the triazine ring. This arrangement together with the tert-butyl-groups protects the Trz rings from contact with small molecules such as O_2_ or H_2_O. Both the phenyl substituted triazine ring and the carbazole substituents show an in-plane arrangement with the same molecular building blocks of neighbouring molecules (Cz in-plane with Cz, Trz in-plane with Trz). Moreover, the phenyl ring and the triazine ring are in-plane, indicating an electronic conjugation between these moieties. In contrast, in the tetrafluorinated DTPT-D2FCz the phenyl ring and the triazine ring are no longer in plane but show a relative tilt of more than 10°. Here the molecular ordering is clearly driven by pairwise π-stacking of the fluorinated carbazole substituents. In this crystal structure the triazine rings are completely unprotected against contact with small electron-trapping molecules such as O_2_ or H_2_O. This again shows that a ‘closed’ stacking geometry is a prerequisite to obtain trap-free transport by shielding the electron transporting core from extrinsic contaminants.

**Fig. 6:**
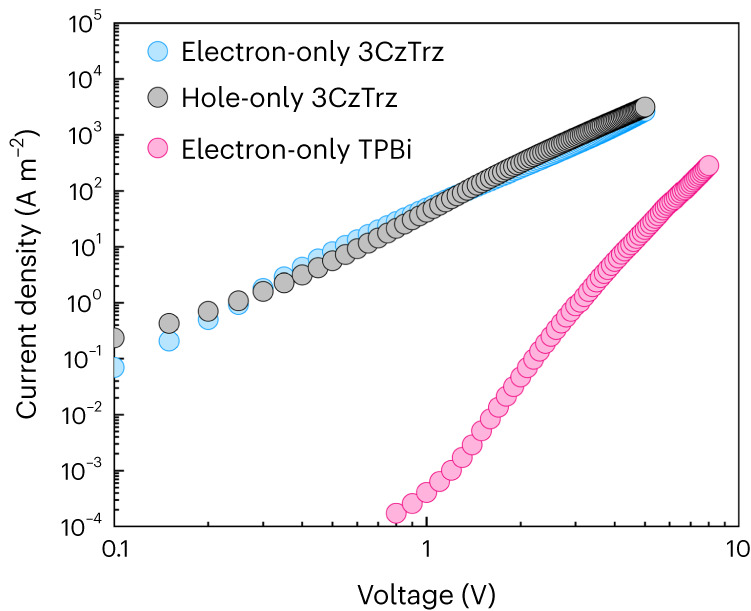
Electron and hole current in 3CzTrz and electron current in TPBi. Current density (*J*)–voltage (*V*) characteristics of electron- and hole-only devices of 3CzTrz and TPBi. The active layer thickness in each device is 100 nm.

To put the trap-free electron transport in 3CzTrz further in perspective, in Fig. 6 the electron and hole current for 3CzTrz are shown, together with the electron current of TPBi, a state-of-the-art electron transport material used in multilayer OLEDs and perovskite-based LEDs^27,28^.

For 3CzTrz both the electron and hole current of 3CzTrz are not only nearly trap-free but also balanced in charge-carrier mobility, which amounts to 2 × 10^-9^ m^2^ V^−^^1^ s^−1^. In terms of electron transport, 3CzTrz clearly outperforms TPBi. Figure 6 shows that, due to trapping, the electron current in TPBi is more than two orders of magnitude lower compared with the trap-free 3CzTrz material.

## Outlook

Due to stack integrity issues, printed blue OLEDs consist preferably of only one or two solution-processed layers. However, the absence of simultaneous trap-free transport of both electrons and holes in large band gap organic semiconductors so far has prevented the realization of efficient single-layer blue OLEDs (3 eV band gap), as trapping has a strong negative effect on their efficiency. The approach presented here shows that by manipulating the molecular structure of donor–acceptor molecules the trapping by defects can be prevented. As a result, this work paves the way towards efficient printed blue OLEDs in future.

## Methods

### Synthesis

1CzTrz–5CzTrz and their fluorinated analogues (1CzTrz-F–3CzTrz-F) as well as DTPT-DCz, DTPT-DFCz and DTPT-D2FCz were synthesized according to procedures in literature and purified by vacuum sublimation. Details are given in the Supplementary Information. TPBi was purchased from Luminescence Technology. Chemicals for synthetic operations were purchased from common suppliers (Sigma-Aldrich, Fisher Scientific, VWR etc.) and were used as received.

### Device fabrication and measurements

Electron-only devices were fabricated on glass substrates. The substrates were cleaned with detergent solution and were ultrasonicated in acetone and isopropyl alcohol. The substrates were heated to 140 °C for 10 min and subsequently treated with ultraviolet–ozone for 20 min. The substrates were transferred into a nitrogen-filled glove box, and 30 nm of Al was thermally evaporated, followed by the organic layer (~100 nm) and a 4 nm TPBi layer. For completion, a 5 nm Ba and 100 nm Al layer was evaporated on top. Electrical characterization was carried out under N_2_ atmosphere with a Keithley 2400 source meter.

### Solution NMR measurements

All solution NMR spectra (^1^H, ^13^C{H}) were measured using a Bruker Avance III setup at 700.25 MHz ^1^H Larmor frequency and were performed at 298 K with deuterated tetrachloroethane if not differently specified. Chemical shift values *δ* are given in parts per million, and coupling constants *J* are given in hertz. The multiplicity of signals is described using the following shortcuts: s (singlet), d (doublet), dd (doublet of doublets), t (triplet), q (quartet) and m (multiplet).

### Solid state NMR measurements

The solid samples were packed into Bruker BioSpin zirconia rotors with 1.3 mm outer diameter. ^1^H MAS NMR spectra were acquired with four scans of direct excitation using a 2.0 µs 90 degree excitation pulse and a recycle delay of 30 s on a Bruker Avance NEO spectrometer operating at 850.27 MHz ^1^H Larmor frequency at a MAS spinning speed of 50 kHz. ^19^F MAS NMR spectra were acquired with 16 scans of direct excitation using a 2.5 µs 90° excitation pulse and a recycle delay of 30 s on a Bruker Avance III spectrometer at 470.61 MHz ^19^F Larmor frequency and 25 kHz MAS spinning frequency.

### Photoelectron spectroscopy

Ionization energies were measured with an atmospheric photoemission yield spectrometer (AC-2) from Riken Keiki Co., Ltd.

### Mass spectrometry

Matrix-assisted laser desorption ionization with time-of-flight analysis was performed on a rapifleX MALDI-ToF/ToF from Bruker. Atmospheric pressure chemical ionization MS was recorded with atmospheric pressure solids analysis probe using an Advion expression compact mass spectrometer.

### Cyclic voltammetry

Cyclic voltammetry was carried out on a computer-controlled GSTAT12 in a three-electrode cell in anhydrous acetonitrile solution of *n*-Bu4NPF6 (0.05 M) with a scan rate of 100 mV s^−1^ at room temperature under argon. Pt wires were used as the counter and working electrodes; a silver wire was applied as the reference electrode.

### Reporting summary

Further information on research design is available in the Nature Portfolio Reporting Summary linked to this article.

## Online content

Any methods, additional references, Nature Portfolio reporting summaries, source data, extended data, supplementary information, acknowledgements, peer review information; details of author contributions and competing interests; and statements of data and code availability are available at 10.1038/s41563-023-01592-3.

## Supplementary information

Supplementary Information Supplementary Figures 1–15, Supplementary Tables 1–15, description of synthesis.

Reporting Summary

## Data Availability

The data that support the plots within this paper and other findings of this study are available in Figshare, 10.6084/m9.figshare.23099051 force fields of DOS calculations, 10.6084/m9.figshare.23099054 current-voltage, cyclic voltammetry, photoluminescence, ultraviolet–visible and UPS of organic compounds, 10.6084/m9.figshare.23099057 NMR and XRD data.
