# Supplementary material for: Elimination of charge-carrier trapping by molecular design
Source: Nat Mater. 2023 Jun 29;22(9):1114–20. doi: 10.1038/s41563-023-01592-3 (PMC10465354; doi:10.1038/s41563-023-01592-3)
Supplement: Supplementary file 1 — Supplementary Figures 1–15, Supplementary Tables 1–15, description of synthesis. [file 41563_2023_1592_MOESM1_ESM.pdf]

nature materials

Article https://doi.org/10.1038/s41563-023-01592-3

Elimination of charge-carrier trapping by molecular design

In the format provided by the authors and unedited

Supplementary information

Table of Content

1. Supporting experimental results p. 2-7
2. Computational simulations p. 7-15
3. Crystallographic data p. 15-16
4. Synthesis p. 16-31
5. References p. 31-34

1. Supporting experimental results

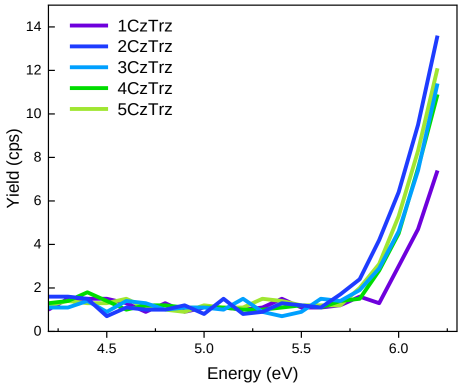

Fig. S1 | Photoemission yield spectroscopy in air of 1–5CzTrz.

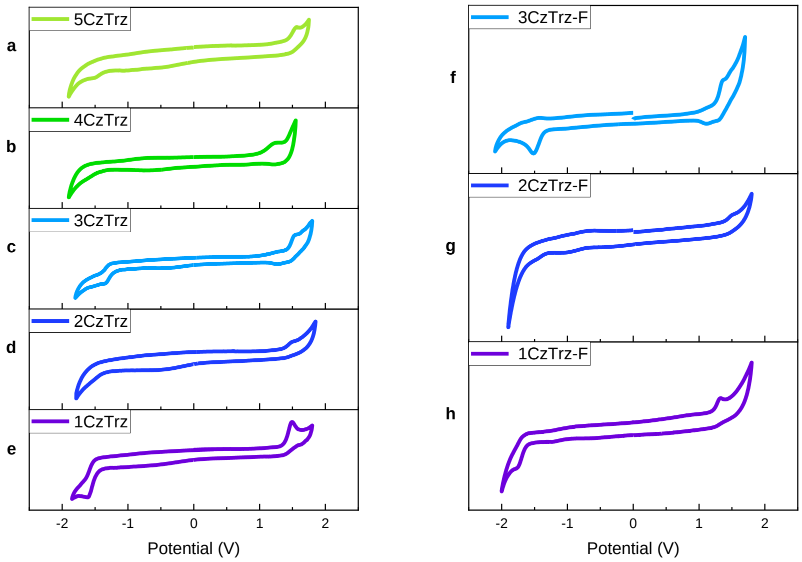

Fig. S2 | Cyclic voltammetry results of 1–5CzTrz and 1–3CzTrz-F. The oxidation potential of Fc/Fc^+ against Ag/AgCl was recorded in acetonitrile-$BuNPF_6$ solution (0.05 M) as 0.34 V. $E = -\left(E_{onset} - E_{\frac{Fc}{Fc^+}} + 4.8\right)$ eV.

Table S1. Experimental ionization energy (IE) (UPS, CV) and electron affinity (EA) (CV).

	**1CzTrz(-F)**	**2CzTrz (-F)**	**3CzTrz (-F)**	**4CzTrz**	**5CzTrz**
IE (UPS) (eV)	5.90(5.95)	5.73(5.56)	5.81(5.90)	5.82	5.81
IE (CV) (eV)	5.82 (5.61)	5.78 (5.80)	5.84 (5.67)	5.43	5.84
EA (CV) (eV)	3.01 (2.84)	3.12 (3.12)	3.28 (3.11)	3.05	3.15

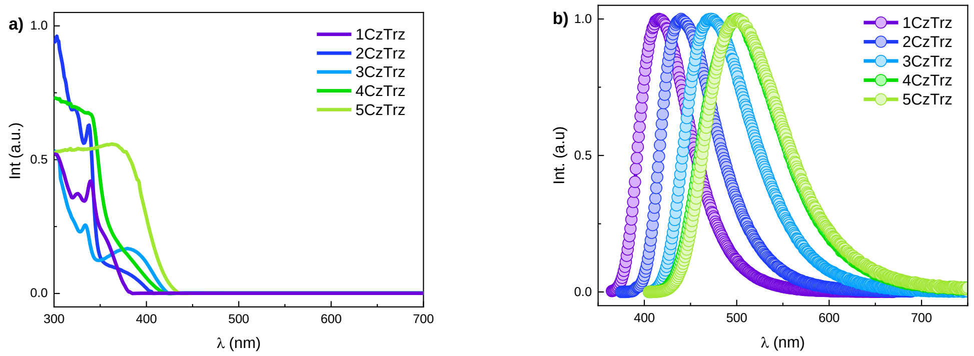

Fig. S3 │ Steady-state UV-Vis spectra a) and photoluminescence spectra b) in solution (toluene) of 1–5CzTrz.

Table S2. Calculated electron trap densities.

	1CzTrz	2CzTrz	3CzTrz	4CzTrz	5CzTrz
Trap density (m^{-3})	6.0×10^{22}	8.0×10^{22}	0.24×10^{22}	6.0×10^{23}	2.8×10^{24}

Variation in injection barrier or built-in voltage V_{bi}?

We demonstrate that the observed difference of orders of magnitude in the electron current cannot be the result of a variation in injection barrier or built-in voltage V_{bi}. First, all compounds tested have relatively similar LUMO levels, such that when using the same injecting cathode only a minor variation in electron injection barrier would be possible. Furthermore, due to the similarity in LUMO energies and the use of identical electrodes also V_{bi} will be similar for all devices. We have realized Ohmic contacts by using a 1,3,5-tris(1-phenyl-1*H*-benzo[d]imidazol-2-yl)benzene, (TPBi) tunnel barrier to decouple the semiconductor from the electrode. The fact that the injecting electron contacts are truly Ohmic is further verified by the thickness dependence of the current.

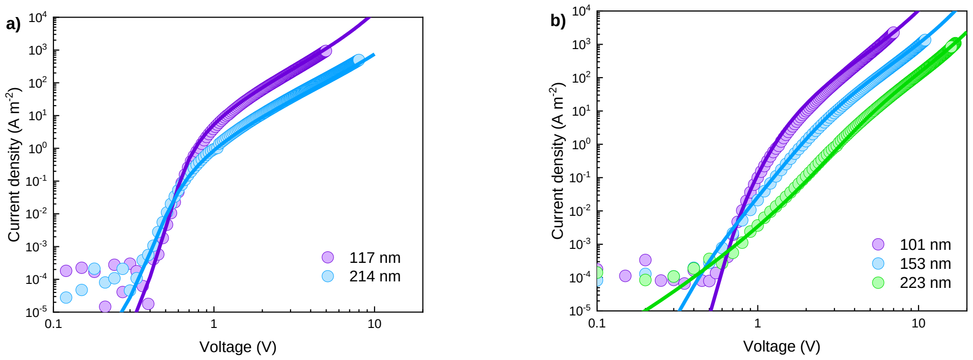

Fig. S4 | Electron transport of 3CzTrz and 2CzTrz with varying thickness. Current density-voltage characteristics of 3CzTrz (a) and 2CzTrz (b) electron-only devices with varying thickness of the semiconductor layer. Symbols are experimental data and solid lines are fits with a numerical drift-diffusion model. Obtained trap densities N_t, trap depths E_t, width of the Gaussian trap distributions σ_t, lattice constants a, DOS variances σ and mobilities μ for electrons are given in the Table S3.

For the nearly trap-free electron current in the **3CzTrz** compound at low voltages the thickness independent diffusion current can be observed (Fig. S4a), showing that V_{bi} in our samples only amounts to 0.4-0.5 V and can therefore not be responsible for the low current at higher voltages as observed for the other compounds. Furthermore, in **3CzTrz** at higher voltages the space-charge limited current with quadratic voltage dependence and thickness dependence to the third power (L^3) is observed, demonstrating that the contact is Ohmic, so there is no injection barrier present. All simulations were performed without an injection barrier. The low current of **2CzTrz** shows the strong thickness dependence as expected for a trap-limited current with Ohmic contacts (Supplementary Fig. S4b). Furthermore, the temperature dependence of the electron current of all **1–5CzTrz** compounds, can be consistently modelled using Ohmic contacts (Supplementary Fig. S5). Similar results have been found for the fluorinated compounds **1-3CzTrz-F**. The electron current in **3CzTrz-F** is space-charge limited and nearly trap-free, whereas for **2CzTrz-F** the current exhibits the characteristic temperature dependence of a trap-limited current with Ohmic contacts (Supplementary Fig. S6). The simulation parameters are listed in Supplementary Table S3 and Table S4 for the **1–5CzTrz** and **1–3CzTrz-F** compounds, respectively. We also verified that our electron J-V characteristics were stable over time (Fig.

S7).

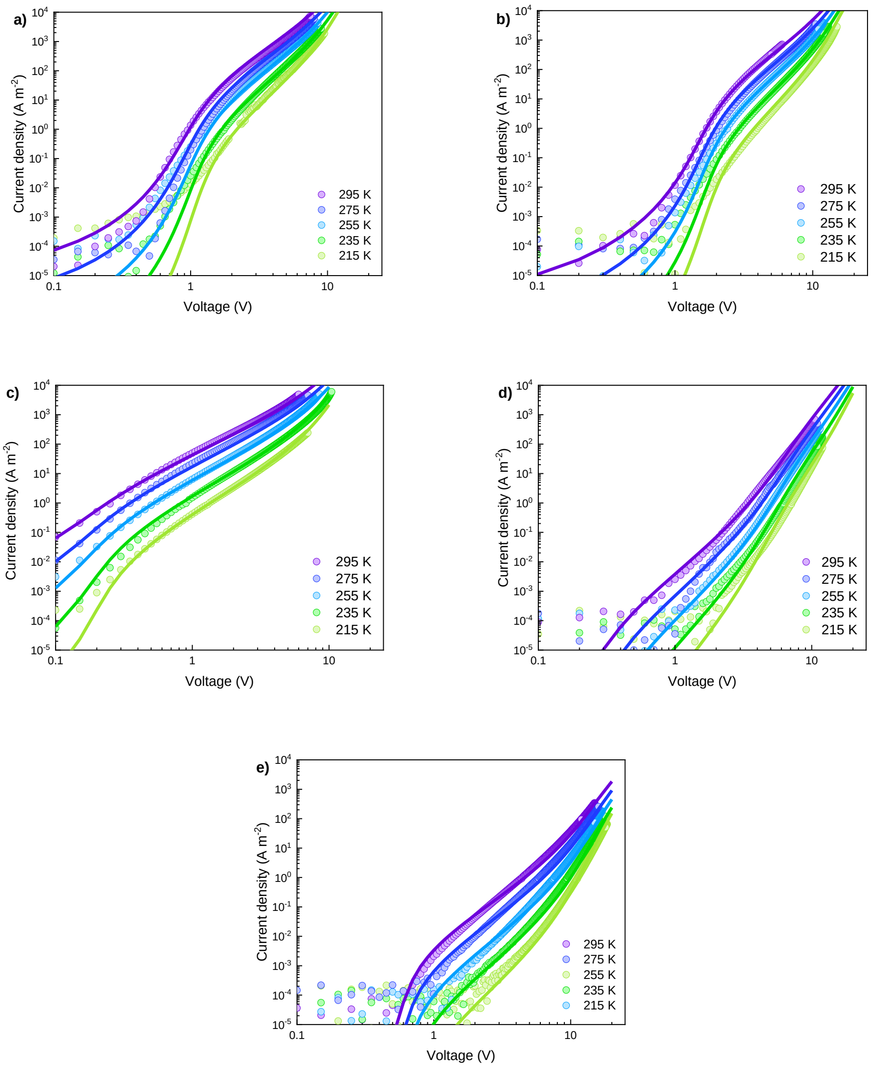

Fig. S5 | Temperature dependent electron transport of 1–5CzTrz. Current density-voltage characteristics of 1CzTrz (94 nm) (a), 2CzTrz (100nm) (b), 3CzTrz (98 nm) (c), 4CzTrz (79nm) (d) and 5CzTrz (102 nm) (e) electron-only devices as function of temperature. Symbols are experimental data and solid lines are fits with a numerical drift-diffusion model. The model parameters are given in Table S3.

Table S3. Drift-diffusion modeling parameters for electron-only devices of 1–5CzTrz.

	**1CzTrz**	**2CzTrz**	**3CzTrz**	**4CzTrz**	**5CzTrz**
Trap density, N_t gauss (× 10^{22} m^{-3})	6.0	8.0	0.24	60	280
Trap depth, E_t (eV)	0.78	0.77	0.70	0.56	0.47
Width of Gaussian trap distribution, σ_t (eV)	0.10	0.10	0.10	0.10	0.08
Lattice constant EGDM, a (× 10^{-9} m)	1.6	1.4	1.5	0.95	1.0

DOS variance EGDM, σ (eV)	0.11	0.12	0.11	0.12	0.11
Mobility at 295 K, μ ($\times 10^{-10}$ m²/Vs)	5.19	1.16	5.19	1.16	5.19

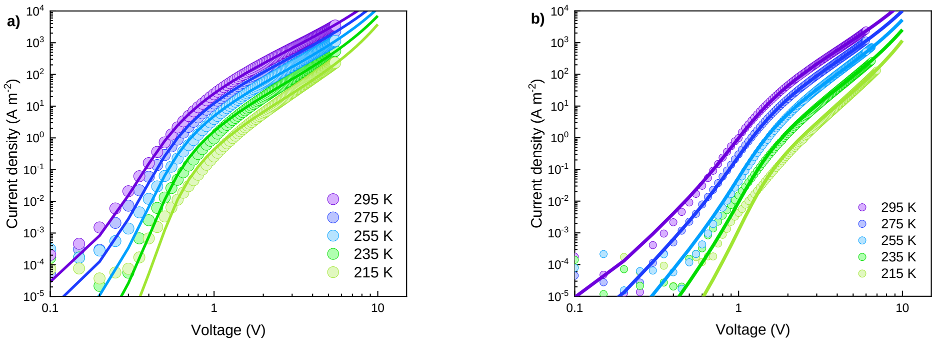

Fig. S6 | Temperature dependent electron transport of 3CzTrz-F and 2CzTrz-F. Current density-voltage characteristics of 3CzTrz-F (118 nm) (a) and 2CzTrz-F (91 nm) (b) electron-only devices as function of temperature. Symbols are experimental data and solid lines are fits with a numerical drift-diffusion model. The model parameters are given in Table S4.

Table S4. Drift-diffusion modeling parameters for electron-only devices of 1–3CzTrz-F.

	**1CzTrz-F**	**2CzTrz-F**	**3CzTrz-F**
Trap density, N_t gauss ($\times 10^{22}$ m^{-3})	100	6.0	0.9
Trap depth, E_t (eV)	0.48	0.63	0.70
Width of Gaussian trap distribution, σ_t (eV)	0.10	0.10	0.10
Lattice constant EGDM, a ($\times 10^{-9}$ m)	1.60	1.40	1.80
DOS variance EGDM, σ (eV)	0.10	0.11	0.09
Mobility at 295 K, μ ($\times 10^{-10}$ m²/Vs)	8.12	4.15	27.92

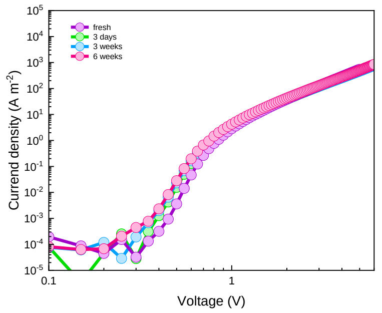

Fig. S7 | Aging of electron-only devices of 3CzTrz. Current density-voltage characteristics of 3CzTrz (155 nm) electron-only devices kept under inert atmosphere and scanned after different aging time (fresh, 3 days, 3 weeks, 6 weeks). No apparent degradation can be observed over aging time.

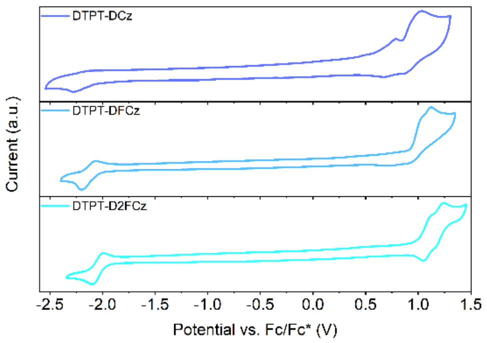

Table S5. Cyclic voltammetry results of DTPT-DCz, DTPT-DFCz and DTPT-D2FCz,

	DTPT-DCz	DTPT-DFCz	DTPT-2FCz
IE (CV) (eV)	-5.49	-5.74	-5.82
EA (CV) (eV)	-2.61	-2.77	-2.81

2. Computer simulations

ω-tuning procedure: To obtain reliable predictions of solid-state IE and EA, we followed the ω-tuning protocol with PCM implicit solvent model ($\varepsilon = 3.0$).[29] The optimal ω was obtained by minimizing the target function

$$J(\omega) = \left|IE_N(\omega) + \epsilon_{N,HOMO}(\omega)\right| + \left|IE_A(\omega) + \epsilon_{A,HOMO}(\omega)\right|$$

where N and A represent the neutral and anionic states, respectively. The computations were performed at the ωB97X-D/def2-TZVP level of theory. The optimal ω for each compound was stored in a database, ranging from 0.009 to 0.024 Bohr^{-1}. This ω range is close to the values reported in the literature.[29,30]

Force field parametrization: All bonded parameters apart from proper and improper dihedrals were taken from the OPLS-AA force field[31,32] and our previous work.[33] The non-bonded parameters, atomic partial charges and Lennard-Jones parameters, were derived following the protocol proposed by Cole *et al.*[34] In short, the overlapping atomic electron densities were obtained via the density-derived electrostatic and chemical (DDEC6) electron density partitioning scheme.[35] The atomic partial charges can then be obtained by integrating the corresponding atomic electron densities over the whole space. Additionally, the two parameters,

A and B, in Lennard-Jones potential are then derived using Tkatchenko−Scheffler (TS) scheme,[36] where the radius of the free atom in a vacuum is taken from reference 6. The electron density was obtained using Gaussian16[37] at ωB97X-D[38]/6-311G(d,p) level and the DDEC6 computations were performed using Chargemol of version 09_26_2017.[35] All molecules considered in this work were partitioned into several rigid fragments following the same procedure as in our previous work.[33] After nonbonded parameters were set, the dihedral potentials that connect these rigid fragments, which are usually missing in the OPLS-AA database, were parameterized using constrained optimization scanning, performed at ωB97X-D3/def2-TZVP level using ORCA 4.2.1.[39] For more details of the parameterization of dihedral potentials, please refer to ref.[40]

MD simulations: All classical MD simulations were performed using GROMACS of version 2020.3.[41,42] For the long-range electrostatic interactions, the particle mesh Ewald (PME) method was employed with a 0.12 nm Fourier spacing. A cutoff of 13 Å was applied to all non-bonded interactions. The temperature and pressure control were accomplished using velocity rescaling with a stochastic term[43] ($\tau_T = 0.5$ ps) and an isotropic coupling for the pressure from a Berendsen barostat ($P_0 = 1$ bar, $\chi = 4.5 \times 10^{-5}$ bar^{-1} , and $\tau_P = 0.5$ ps).

For each compound, 2000 molecules were initially randomly placed in a simulation box with a low target density around 200 kg/m^3 using Packmol.[44] The whole system was then heated up from 100K to 300K at a rate of 0.67 K/ps. It was then equilibrated at 300 K until the density reached a steady value. This step helps to prevent the system from exploding due to a high heating rate. Finally, the system was heated up from 300 K to 800 K at a rate of 0.5 K/ps, followed by an equilibration at 800 K for 10 ns. The equilibration time is long enough to ensure a steady density of the system for all compounds discussed here. Finally, the system underwent a linear cooling procedure from 800 K to 300 K at a 500 K/ns cooling rate followed by an equilibration at 300 K for 9 ns.

DOS calculations: To compute the density of states (DoS) of the triazine materials and oxygen impurities, oxygen molecules were inserted into the equilibrated amorphous structure of the triazine material,*NTz = 2000*. The oxygen impurities were introduced at random positions within the amorphous structure, with random orientation, using GROMACS at a concentration of *NO2 = 100*, corresponding concentrations are given in Table S6.[41]

Table S6. Concentration of interstitial oxygen gas in in amorphous triazine materials.

	**1CzTrz-F**	**2CzTrz-F**	**3CzTrz-F**	**4CzTrz**	**5CzTrz**
Interstitial oxygen conc. (mmol dm^{-3})	112.9	91.0	71.8	60.8	52.5

The systems were then energy minimized and equilibrated for 1 ns at $T = 300\ K$ in the NPT ensemble. DoS computations were performed using the VOTCA package and used a perturbative scheme which evaluates the difference in system energies when molecules are present in their neutral or anionic form.[45–51] In this work we consider electrostatic, E_{stat}, and induction energy, E_{indu}, components to the total site energy in order to evaluate a solid state correction, $\Delta EA_s = E_{\text{stat}} + E_{\text{indu}}$, to the gas phase electron affinity, EA_0. This is used to estimate the bulk electron affinity at each site, $EA_{\text{bulk}} = EA_0 + \Delta EA_s$.[5] The electrostatic contribution is calculated by evaluating the Coulombic sums of atom centered multipoles. The multipoles were calculated using distributed multipole analysis with the GDMA program and were obtained for both the neutral and anionic ground states of oxygen and the triazine molecules. The multipole expansion of each molecule is evaluated by matching the molecular polarizability tensor with the equivalent polarizability tensor computed using GAUSSIAN 16.[37] For the interstitial oxygen this was done using the aug-cc-pvqz basis set and CCSD(T) while the triazine molecules were evaluated using DFT with the B3LYP functional and the 6-311G+(d, p) basis set.

The inductive component is evaluated using a polarizable force field, based on the Thole model, which utilizes isotropic atomic polarizabilities for each atom *a* on molecule *i*, *αa, i*. The polarizable force field based off the Thole model utilizes fixed atomic polarizabilities for each element, derived from the polarizabilities of the atomic elements. These atomic polarizabilities are then rescaled to reproduce the molecular polarization volume obtained from quantum mechanics calculations, and used to calculate the interaction energy between induced molecular multipoles and the permanent multipoles obtained from GDMA. The molecular polarization volumes were obtained with DFT using the PBE functional and the 6-311G(d, p) basis set using GAUSSIAN16.[52]

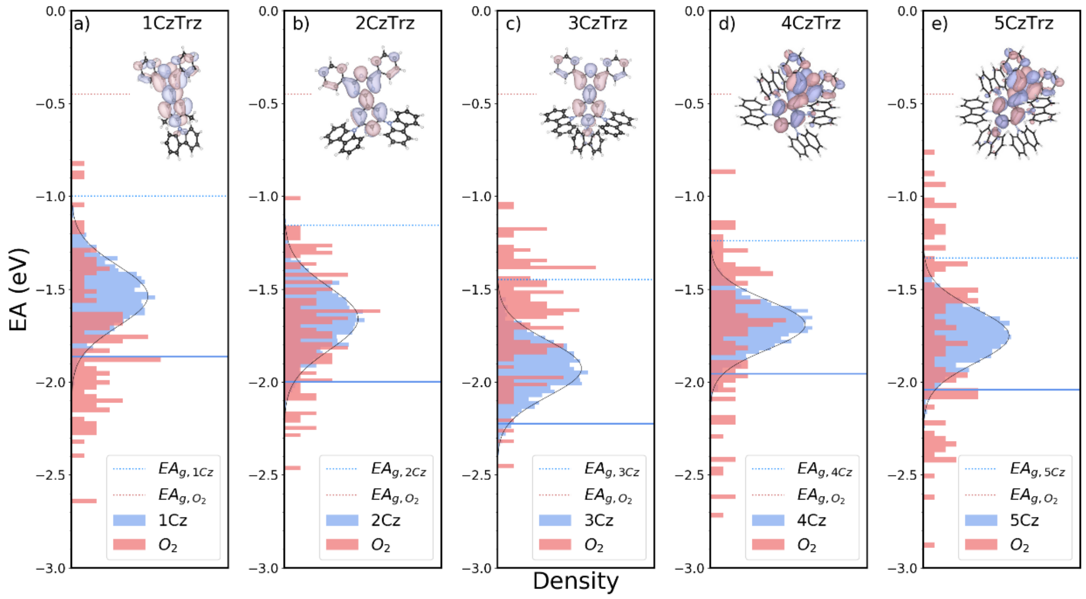

Fig. S8 | Calculated density-of-states distributions for amorphous 1–5CzTrz. The density of states of electron affinity of molecular oxygen (red) and organic materials (blue; **1CzTrz**, **2CzTrz**, **3CzTrz**, **4CzTrz**, and **5CzTrz**) with amorphous morphology. The dotted lines are the gas-phases electron affinity for molecular oxygen and organic materials. The blue solid line represents the energy μ_e -2σ_e, with μ_e corresponding to the average of the calculated solid-state EA values and σ_e the standard deviation of the Gaussian distribution, respectively (Table S7). The energy μ_e -2σ_e is expected to correspond to the onset of the solid-state EA from UPS measurements.

We note that in the representation of the DOS distributions the conventional sign convention for energy band diagrams is used, meaning negative numbers on the energy axis. In this graphical presentation, higher EAs are represented by more negative numbers, implying that electron traps are located below the molecular DOS at more negative energies. Apart from the presentation in the figures, EA and IE are positive numbers representing the absolute value of the energy with regard to vacuum (zero energy).

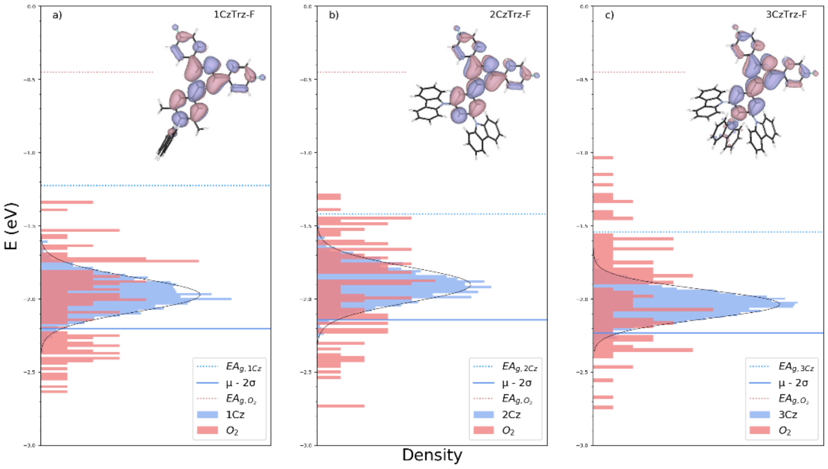

Fig. S9 | Calculated density-of-states distributions for amorphous 1–3CzTrz-F. The density of states of electron affinity of molecular oxygen (red) and organic materials (blue; 1CzTrz-F, 2CzTrz-F and 3CzTrz-F) with amorphous morphology. The dotted lines are the gas-phases electron affinity for molecular oxygen and organic materials. The blue solid line represents the energy μ_e -2σ_e, with μ_e corresponding to the average of the calculated solid-state EA values and σ_e the standard deviation of the Gaussian distribution, respectively (Table S9), The energy μ_e -2σ_e is expected to correspond to the onset of the solid-state EA from UPS measurements.

Table S7. **Material properties in amorphous phase of 1–5CzTrz.**

	**1CzTrz-F**	**2CzTrz-F**	**3CzTrz-F**	**4CzTrz**	**5CzTrz**
μ_e (eV)	-1.971	-1.903	-2.036	-1.692	-1.751
σ_e (eV)	0.115	0.119	0.098	0.132	0.146
EA (eV)	-2.201	-2.131	-2.233	-1.955	-2.042
μ_h (eV)	-5.879	-6.085	-5.900	-5.749	-5.735
σ_h (eV)	0.210	0.113	0.139	0.149	0.162
IE (eV)	-5.459	-5.858	-5.622	-5.450	-5.411

Table S8. Oxygen trap levels in amorphous 1–5CzTrz.

	**1CzTrz-F**	**2CzTrz-F**	**3CzTrz-F**	**4CzTrz**	**5CzTrz**
μ_e (eV)	-2.014	-1.916	-1.942	-1.706	-1.745
σ_e (eV)	0.294	0.315	0.340	0.365	0.431
EA (eV)	-2.602	-2.546	-2.622	-2.436	-2.607

Table S9. **Material properties in amorphous phase of 1–3CzTrz-F.**

	**1CzTrz-F**	**2CzTrz-F**	**3CzTrz-F**
μ_e (eV)	-1.971	-1.903	-2.036

σ_e (eV)	0.115	0.119	0.098
EA (eV)	-2.201	-2.131	-2.233
μ_h (eV)	-5.879	-6.085	-5.900
σ_h (eV)	0.210	0.113	0.139
IE (eV)	-5.459	-5.858	-5.622

Table S10. Oxygen trap levels in amorphous 1–3CzTrz-F.

	1CzTrz-F	2CzTrz-F	3CzTrz-F
μ_e (eV)	-2.014	-1.916	-1.942
σ_e (eV)	0.294	0.315	0.340
EA (eV)	-2.602	-2.546	-2.622

Table S11. Material properties in crystalline phase of 1CzTrz-F and 3CzTrz-F.

	1CzTrz-F	3CzTrz-F
μ_e (eV)	-2.065	-2.031
σ_e (eV)	0.066	0.067
EA (eV)	-2.197	-2.166
μ_h (eV)	-6.028	-5.913
σ_h (eV)	0.099	0.083
IE (eV)	-5.831	-5.746

Table S12. Oxygen trap levels in crystalline phase of 1CzTrz-F and 3CzTrz-F.

	1CzTrz-F	3CzTrz-F
μ_e (eV)	-2.503	-1.900
σ_e (eV)	0.227	0.346
EA (eV)	-2.958	-2.593

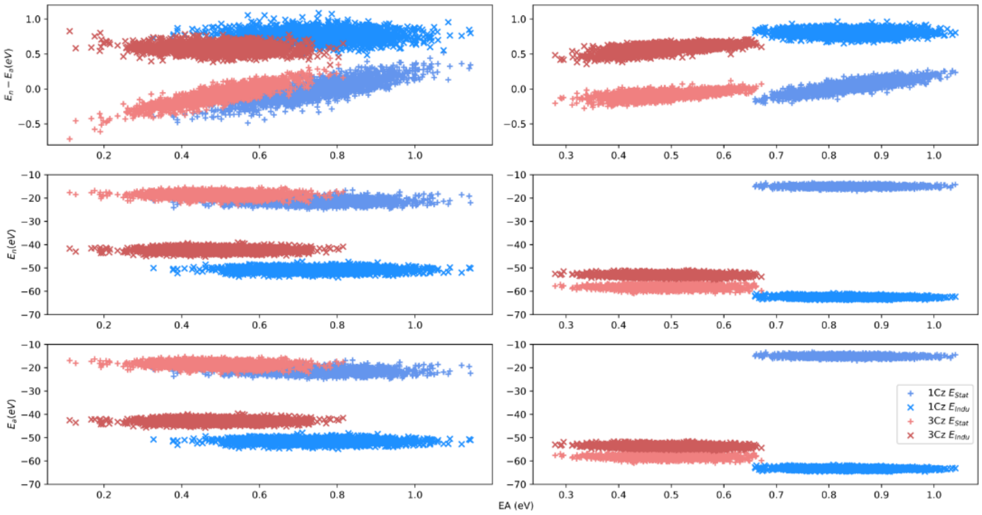

Fig. S10 │ Static and inductive electronic energy contributions to site energies of **1CzTrz-F** and **3CzTrz-F** in amorphous, 1st column and crystalline phases, 2nd column.

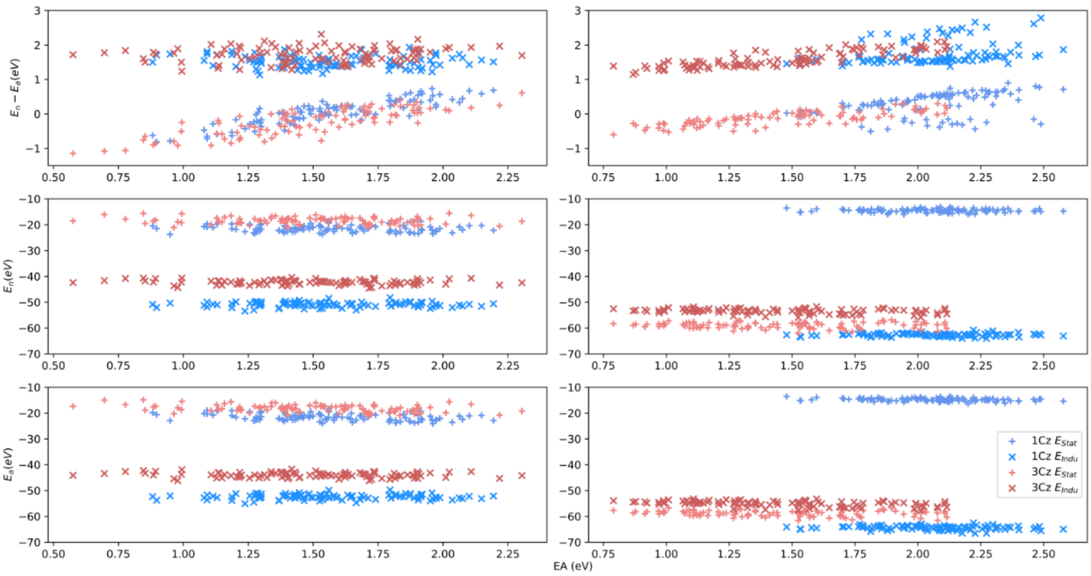

Fig. S11 │ Static and inductive electronic energy contributions to site energies of interstitial oxygen in amorphous, 1st column, and crystalline, 2nd column, **1CzTrz-F** and **3CzTrz-F**

Table S13. Standard deviation of correlated and uncorrelated site energy distributions, with no interstitial oxygen, in crystalline **1CzTrz-F** and **3CzTrz-F** systems.

	Uncorrelated σ_e (eV)	Correlated σ_e/sqrt(2) (eV)
**1CzTrz-F**	0.06065	0.07298
**3CzTrz-F**	0.06573	0.10795

Additional calculations to obtain the correlated EA distribution of 1CzTrz-F and 3CzTrz-F in the solid state were also performed. These found a positive correlation of site energy distributions in **1CzTrz-F** (Figure S12 and S13, Table S13), showing that the site energies are dependent on those of the neighboring sites, further supporting the conclusion that the difference in experimentally observed trap density between **1CzTrz-F** and **3CzTrz-F** is likely to be attributed to differences in morphologies.

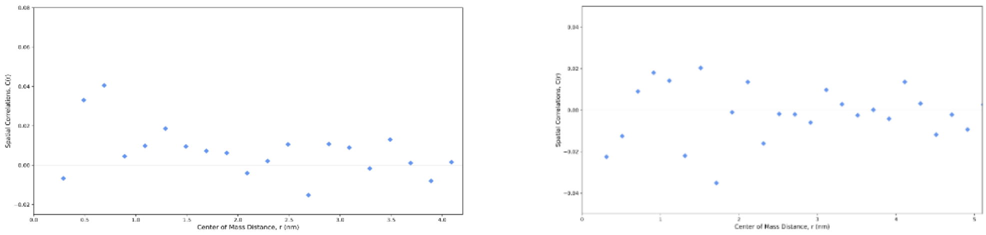

Fig. S12 │ Spatial site energy correlations, with interstitial oxygen, in crystalline (a) **1CzTrz-F** and (b) **3CzTrz-F.**

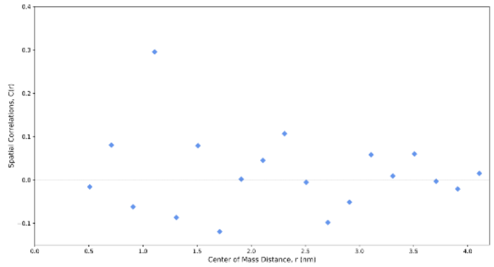

Fig. S13 │ Spatial site energy correlations of crystalline (a) **1CzTrz-F** and (b) **3CzTrz-F** with no interstitial oxygen.

Table S14. Drift-diffusion modeling parameters for electron-only devices of DTPT-DCz, DTPT-DFCz and DTPT-D2FCz.

	**DTPT-DCz**	**DTPT-DFCz**	**DTPT-D2FCz**
Trap density, N_t gauss ($\times 10^{22}$ m^{-3})	1.5	23	65
Trap depth, E_t (eV)	0.74	0.80	0.70
Width of Gaussian trap distribution, σ_t (eV)	0.12	0.139	0.12
Lattice constant EGDM, a ($\times 10^{-9}$ m)	1.3	1.4	1.1
DOS variance EGDM, σ (eV)	0.12	0.139	0.12
Mobility at 295 K, μ ($\times 10^{-11}$ m²/Vs)	3	0.63	1.5

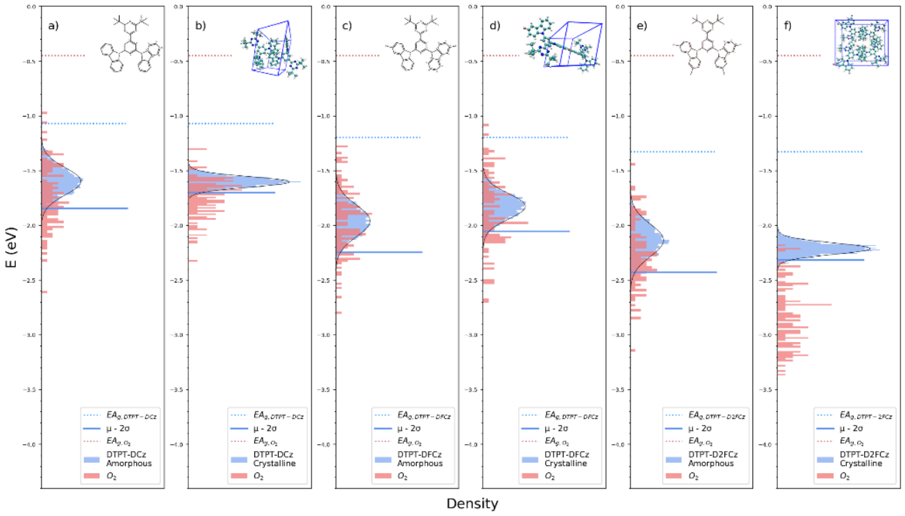

Fig. S14│ Calculated density-of-states distributions. a,b,c,d,e,f The density of states of electron affinity of (**a**) amorphous **DTPT-DCz**, (**b**) crystalline **DTPTDCz**, (**c**) amorphous **DTPT-DFCz**, (**d**) crystalline **DTPT-DFCz**, (e) amorphous **DTPT-D2FCz** and (f) crystalline **DTPT-D2FCz**, respectively.

3. Crystallographic data

Table S15. Crystallographic results of single crystal XRD. Crystallographic parameters for the compounds 1CzTrz, 1CzTrz-F and 3CzTrz-F.

	**1CzTrz**	**1CzTrz-F**	**3CzTrz-F**
Space Group	P 2_1/c	P 2_1/c	P $\bar{1}$
Cell Lengths	**a** 24.701(3)<br>**b** 11.8995(8)<br>**c** 8.8160(9)	**a** 25.2325(7)<br>**b** 12.3048(3)<br>**c** 8.5631(2)	**a** 13.7364(4)<br>**b** 16.4858(6)<br>**c** 21.1482(7)
Cell Angles	α 90<br>β 92.100(8)<br>γ 90	α 90<br>β 93.375(2)<br>γ 90	α 80.448(3)<br>β 78.790(2)<br>γ 70.435(3)
Cell Volume	2589.55	2654.07	4400.09

Solid-state NMR data

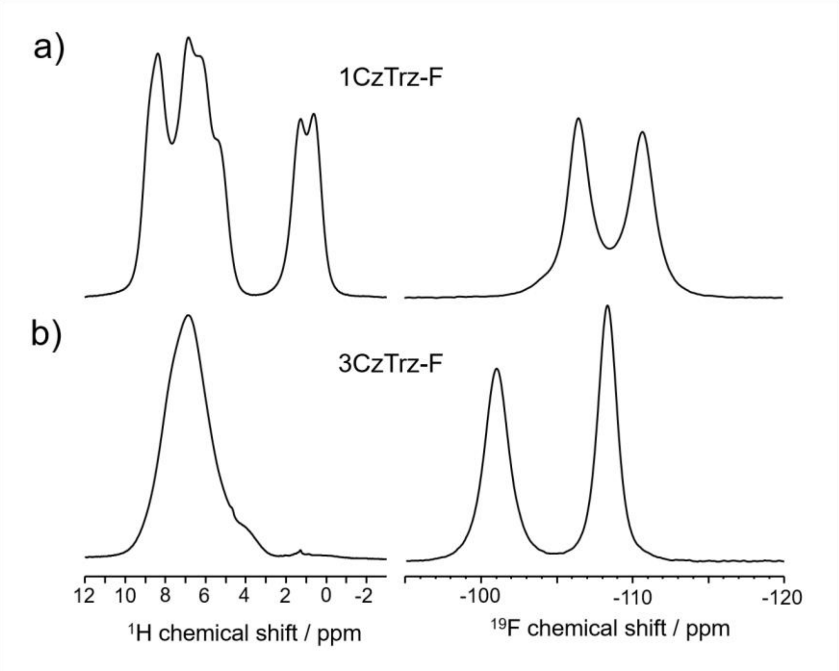

Fig. S15 | NMR spectra of the 1CzTrz-F and 3CzTrz-F compounds. a,b, ^{1}H and ^{19}F MAS spectra recorded at 50 kHz spinning speed of vapor deposited material of the compounds **1CzTrz-F** (a) and **3CzTrz-F** (b).

4. Synthesis

1st series: Preparation of 1CzTrz(-F), 2CzTrz(-F), 3CzTrz(F-), 4CzTrz and 5CzTrz.

General procedure for the introduction of carbazole.[53] A mixture of carbazole (3 Cz: 3 eq., 2 Cz: 2 eq., 1 Cz: 0.5 eq.) and Cs_2CO_3 (3 Cz: 4 eq., 2 Cz: 2.5 eq., 1 Cz: 5.0 eq.) was placed in a two necked flame-dried flask under an argon atmosphere, suspended in DMF and stirred at room temperature for 30 min, followed by addition of corresponding fluorobenzene (3 Cz: 1 eq., 2 Cz: 1 eq., 1 Cz: 2 eq.) and heating to 175 °C for 24 h (1 Cz: 72h). After cooling to room temperature, the reaction mixture was poured on water and the water phase was extracted three times with dichloromethane (DCM). The combined organic phases were washed with brine solution and dried over $MgSO_4$. The solvent was evaporated under reduced pressure and the crude product was purified by column chromatography (stationary phase: silica, eluent: hexane/DCM 4:1).

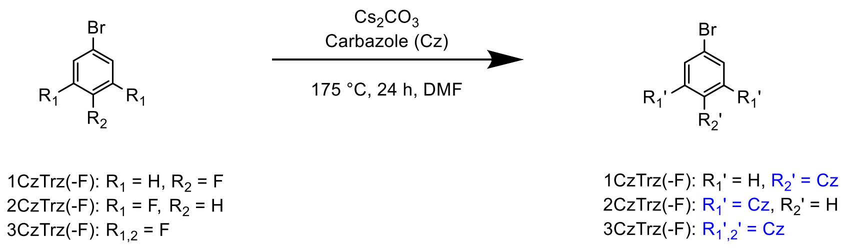

9-(4-bromo-2,6-dimethylphenyl)-9*H*-carbazole

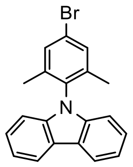

According to the general procedure 5-bromo-2-fluoro-1,3-dimethylbenzene (2.43 g, 12.0 mmol), carbazole (1.01 g, 6.0 mmol) and Cs_2CO_3 (10.50 g, 30.0 mmol) were suspended

in DMF (30 mL). The purified product was obtained as a white solid (1.69 g, 81%). ^{1}H NMR [ppm] (700 MHz, $C_2D_2Cl_4$): 8.16 (H^3, d, $J^3_{H\text{-}H}$=7.7 Hz, 2H), 7.44 (H^2, s, 2H), 7.39 (H^1 ,t, $J^3_{H\text{-}H}$ = 7.7 Hz, 2H), 7.28 (H^2 ,t , $J^3_{H\text{-}H}$ =7.7 Hz, 2H), 6.93 (H^6 ,d , $J^3_{H\text{-}H}$ = 8 Hz, 2H), 1.82 (H^{11} , s, 6H). ^{13}C NMR [ppm] (176 MHz, $C_2D_2Cl_4$): 140.4, 140.1, 133.8, 131.7, 126.3, 123.0, 122.5, 120.6, 119.8, 109.5, 17.5. APCI calc. for $C_{20}H_{16}BrN$ 349.05, found 350.20.

9,9'-(5-bromo-1,3-phenylene)bis(9*H*-carbazole)

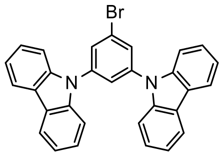

According to the general procedure 1-bromo-3,5-difluorobenzene (0.50 g, 2.6 mmol), carbazole (0.87 g, 5.2 mmol) and Cs_2CO_3 (2.53 g, 7,8 mmol) were suspended in DMF (20 mL). The purified product was obtained as a white solid (0.85 g, 67%). ^{1}H NMR [ppm] (700 MHz, $C_2D_2Cl_4$): 8.17 (d, J^3 = 7.4 Hz, 4H), 7.89 (d. J^4 = 1.5 Hz, 2H), 7.81 (t, J^4 = 1.5 Hz, 1H), 7.59 (d, J^3 = 7.9 Hz, 4H), 7.49 (t, J^3 = 6.7 Hz, 4H), 7.36 (t, J^3 = 6.7 Hz, 4H). ^{13}C NMR [ppm] (176 MHz, $C_2D_2Cl_4$): 140.4, 140.2, 128.6, 126.6, 124.4, 123.7, 121.0, 120.7, 109.8. APCI calc. for $C_{30}H_{19}BrN_2$ 486.07, found 486.90.

9,9',9''-(5-bromobenzene-1,2,3-triyl)tris(9*H*-carbazole)

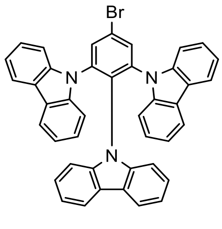

According to the general procedure 5-bromo-1,2,3-trifluorobenzene (1.40 g, 6.6 mmol), carbazole (3.33 g, 19.9 mmol) and Cs_2CO_3 (8.64 g, 26.6 mmol) were suspended in DMF (35 mL). The purified product was obtained as a white solid (2.77 g, 64%). ^{1}H NMR [ppm] (700 MHz, $C_2D_2Cl_4$): 8.1 (d, J^3 = 5.2 Hz, 2H), 7.8 (s, 2H), 7.4 (d, J^3 = 6.8 Hz, 4H), 7.2 (d, J^3 = 7.3 Hz, 4H), 7.1-7.0 (m, 8H), 6.9 (t, J^3 = 7.6 Hz, 2H), 6.8 (t, J^3 = 6.8 Hz, 2H), 6.7 (t, J^3 = 7.0 Hz, 2H). ^{13}C NMR [ppm] (700 MHz, $C_2D_2Cl_4$): 139.7, 138.5, 138.3, 132.8, 131.5, 125.7, 124.9, 123.9, 122.2, 120.5, 120.1, 119.4, 110.0, 109.9. APCI calc. for $C_{42}H_{26}BrN_3$ 651.13, found

651.80.

General procedure for preparation of boronic acid pinacol esters.[54] Under an argon atmosphere the bromine substituted aryl compound (1.0 eq) was dissolved together with KOAc (3.2 eq), bis(pinacolato)diboron (1.3 eq) and Pd(dppf)Cl_2 (3.4 mol%) in 1,4-dioxane. The reaction mixture was stirred over night at 110 °C. After cooling to room temperature, the reaction mixture was washed with water. The water phase was extracted three times with DCM. The combined organic phases were dried over $MgSO_4$ and purified by column chromatography (stationary phase: silica, eluent: hexane/ethyl acetate 95:5).

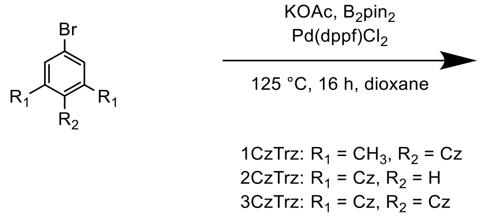

<u>**9,9'-(5-(4,4,5,5-tetramethyl-1,3,2-dioxaborolan-2-yl)-1,3-phenylene)bis(9*H*-carbazole)**</u>

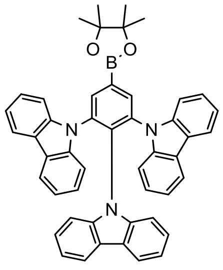

According to the general procedure 9,9',9''-(5-bromobenzene-1,2,3-triyl)tris(9*H*-carbazole) (2.60 g, 3.96 mmol), KOAc (1.24 g, 12.67 mmol), bis(pinacolato)diboron (1.31 g, 5.15 mmol) and Pd(dppf)Cl_2 (95 mg 0.17 mmol) were dissolved in 1,4-dioxane (40 mL). The purified product was obtained as a beige solid (1.40 g, 51%). ^{1}H NMR [ppm] (700 MHz, $C_2D_2Cl_4$): 8.29 (s, 2H), 7.81-7.76 (m, 4H), 7.36 (d, J^3 = 8.8 Hz, 2H), 7.25-7.20 (m, 4H), 7.11-7.00 (m, 10H) 6.76 (t, J^3 = 7.0 Hz, 2H), 6.68 (t, J^3 = 7.0 Hz, 2H), 1.37 (s, 12H). ^{13}C NMR [ppm] (176 MHz, $C_2D_2Cl_4$): 140.2, 138.7, 136.8, 136.1, 134.5, 125.5, 124.7, 123.5, 120.0, 119.9, 119.8, 119.3, 110.3, 110.1, 84.7, 25.1 APCI calc. for $C_{48}H_{38}BN_3O_2$ 699.31, found 699.80.

<u>**9,9'-(5-(4,4,5,5-tetramethyl-1,3,2-dioxaborolan-2-yl)-1,3-phenylene)bis(9*H*-carbazole)**</u>

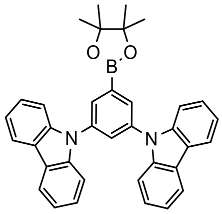

According to the general procedure 9,9'-(5-bromo-1,3-phenylene)bis(9*H*-carbazole) (6.00 g, 12.31 mmol), KOAc (3.87 g, 39.39 mmol), bis(pinacolato)diboron (4.06 g, 16.00 mmol) and Pd(dppf)Cl_2 (310 mg 0.42 mmol) were dissolved in 1,4 dioxane (80 mL). The purified product was obtained as a beige solid (5.11 g, 78%). ^{1}H NMR [ppm] (700 MHz, $C_2D_2Cl_4$): 8.16 (d, J^3 = 7.8 Hz, 4H), 8.10 (d, J^4 = 1.8 Hz, 2H), 7.87 (t, J^4 = 1.8 Hz, 1H), 7.54 (d, J^3 = 7.7 Hz, 4H), 7.46 (t, J^3 = 7.0 Hz, 4H), 7.32 (t. J^3 = 7.0 Hz, 4H), 1.36 (s, 12H). ^{13}C NMR [ppm] (176 MHz, $C_2D_2Cl_4$): 140.7, 138.8, 132.0, 127.7, 126.4, 123.5, 120.6, 120.4, 109.9, 84.6, 25.0. APCI calc. for $C_{36}H_{31}BN_2O_2$ 534.25, found 535.0.

9-(2,6-dimethyl-4-(4,4,5,5-tetramethyl-1,3,2-dioxaborolan-2-yl)phenyl)-9*H*-carbazole

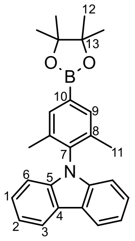

According to the general procedure 9-(4-bromo-2,6-dimethylphenyl)-9*H*-carbazole (1.00 g, 2.86 mmol), KOAc (0.90 g, 9.13 mmol), bis(pinacolato)diboron (0.94 g, 3.17 mmol) and Pd(dppf)Cl_2 (71 mg, 0.10 mmol) were dissolved in 1,4-dioxane (10 mL). The purified product was obtained as a beige solid (0.75 g, 66%). ^{1}H NMR [ppm] (700 MHz, $C_2D_2Cl_4$): 8.14 (H^3, d, J^3_{H-H}=7.7 Hz, 2H), 7.69 (H^2, s, 2H), 7.36 (H^1 ,t, J^3_{H-H} = 7.7 Hz, 2H), 7.25 (H^2 ,t , J^3_{H-H} =7.7 Hz, 2H), 6.89 (H^6 ,d , J^3_{H-H}= 8 Hz, 2H), 1.85 (H^{11} , s, 6H), 1.36 (H^{12}, s, 12H). ^{13}C NMR [ppm] (176 MHz, $C_2D_2Cl_4$): 141.5, 138.6, 138.5, 136.5, 127.5, 124.2, 121.9, 120.9, 111.0, 85.5, 26.5, 18.7. APCI calc. for $C_{26}H_{26}BNO_2$ 397.22, found: 398.3.

General procedure for the preparation of the triazine based semiconductors.[55] Under an argon atmosphere the boronic acid pinacol ester (1.0 eq) was placed together with the

corresponding triazine (1.1 eq.), K_2CO_3 (3.0 eq.) and $Pd(PPh_3)_4$ (5 mol%) in a flame-dried flask and dissolved in a THF/water (2:1) mixture. The solution was heated to 85 °C over night. After cooling down to room temperature the water phase was extracted with DCM for three times (for 2CzTrz based materials see detailed description, due to limited solubility). The combined organic phases were washed with brine solution and dried over $MgSO_4$. The product was purified by column chromatography (stationary phase: silica, eluent: hexane/DCM 4:1) in a first step. The product was further purified by sublimation for device fabrication and solid state measurements.

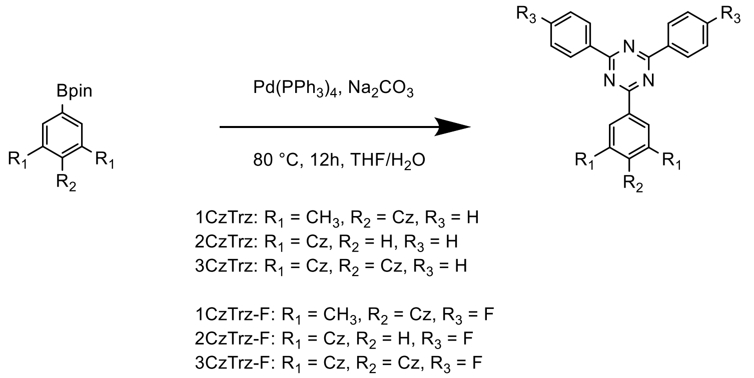

9,9',9''-(5-(4,6-diphenyl-1,3,5-triazin-2-yl)benzene-1,2,3-triyl)tris(9*H*-carbazole) (3CzTrz)

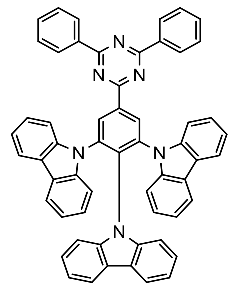

9,9'-(5-(4,4,5,5-tetramethyl-1,3,2-dioxaborolan-2-yl)-1,3-phenylene)bis(9*H*-carbazole) (0.27 g, 1.02 mmol), 2-chloro-4,6-diphenyl-1,3,5-triazine (0.65 g, 0.93 mmol), K_2CO_3 (0.38 g, 2.78 mmol) and $Pd(PPh_3)_4$ (54 mg, 0.05 mmol) were used and dissolved in THF/water (10:5 mL). The purified product was obtained as a pale yellow solid (0.65 g, 94%). ^{1}H NMR [ppm] (700 MHz, $C_2D_2Cl_4$): 9.29 (s, 2H), 8.74 (d, J^3 = 8.4 Hz, 4H), 7.81 (d, J^3 = 8.4 Hz, 4H), 7.61 (t, J^3 = 7.2 Hz, 2H), 7.54 (t, J^3 = 7.3 Hz, 4H), 7.36 (d, J^3 = 8.2 Hz, 2H), 7.32 (d, J^3 = 8.3 Hz, 4H), 7.12-7.05 (m, 8H), 6.99 (d, J^3 = 8.3 Hz, 2H), 6.78 (t, J^3 = 7.2 Hz, 2H), 6.68 (t, J^3 =

7.3 Hz, 2H). ^{13}C NMR [ppm] (176 MHz, $C_2D_2Cl_4$): 172.2, 169.9, 139.9, 138.4, 138.2, 137.7, 135.7, 135.6, 133.2, 130.2, 129.3, 129.0, 125.7, 124.7, 123.7, 123.4, 120.3, 120.0, 119.9, 119.4, 110.3, 110.0. MALDI calc. for $C_{57}H_{36}N_6$ 804.30, found 804.30.

<u>9,9',9''-(5-(4,6-bis(4-fluorophenyl)-1,3,5-triazine-2-yl)benzene-1,2,3-triyl)tris(9*H*-carbazole) (3CzTrz-F)</u>

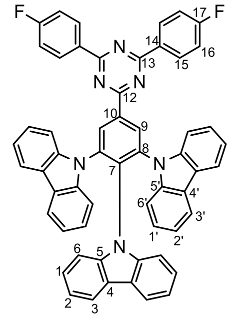

According to the general procedure 9,9'-(5-(4,4,5,5-tetramethyl-1,3,2-dioxaborolan-2-yl)-1,3-phenylene)bis(9*H*-carbazole) (0.65 g, 0.93 mmol), 2-chloro-4,6-bis(4-fluorophenyl)-1,3,5-triazine (0.31 g, 1.02 mmol), K_2CO_3 (0.38 g, 6.39 mmol) and $Pd(PPh_3)_4$ (54 mg, 0.05 mmol) were used and dissolved in THF/water (10:5 mL). The purified product was obtained as a pale yellow solid (0.687 g, 88%). ^{1}H NMR [ppm] (700 MHz, $C_2D_2Cl_4$): 9.23 (H^9, s, 2H), 8.73 (H^{15}, d, J^3 = 8.3 Hz, 4H), 7.81 (H^3, d, J^3 = 8.3 Hz, 4H), 7.35 ($H^{3'}$, d, J^3 = 8.3 Hz, 2H), 7.29 (H^6, d, J^3 = 8.1 Hz, 4H), 7.20 (H^{16}, t, J^3 = 8.2 Hz, 4H), 7.08 (H^2, t, J^3 = 7.2 Hz, 4H), 7.06 (H^1, t, J^3 = 7.3 Hz, 4H), 6.97 ($H^{6'}$, t, J^3 = 8.4 Hz, 2H), 6.77 ($H^{2'}$, 7, J^3 = 7.2 Hz, 2H), 6.66 ($H^{1'}$, t, J^3 = 7.2 Hz, 2H. ^{13}C NMR [ppm] (176 MHz, $C_2D_2Cl_4$): 171.0 (C^{13}), 170.0 (C^{14}), 165.9 (C^{17}), 139.9 (C^5), 138.1 (C^7), 138.0 ($C^{5'}$), 137.7 (C^8), 135.7 (C^{10}), 131.7 (C^{15}), 130.2 (C^9), 125.7 (C^2), 124.9 ($C^{1'}$), 123.7 ($C^{4'}$), 123.4 (C^4), 120.6 ($C^{2'}$), 120.4 (C^1), 119.9 (C^3), 119.3 ($C^{3'}$), 116.0 (C^{16}), 110.2 ($C^{6'}$), 110.0 (C^6). MALDI calc. for $C_{57}H_{34}F_2N_6$ 840.28, found 840.39.

<u>9,9',9''-(5-(4,6-diphenyl-1,3,5-triazin-2-yl)benzene-1,2,3-triyl)tris(9*H*-carbazole)</u>

(2CzTrz)

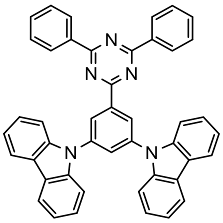

According to the general procedure 9,9'-(5-(4,4,5,5-tetramethyl-1,3,2-dioxaborolan-2-yl)-1,3-phenylene)bis(9*H*-carbazole) (0.70 g, 1.30 mmol), 2-chloro-4,6-diphenyl-1,3,5-triazine (0.39 g, 1.44 mmol), K_2CO_3 (0.54 g, 3.90 mmol) and $Pd(PPh_3)_4$ (75 mg, 0.06 mmol) were used and dissolved in THF/water (10:5 mL). After completion of the reaction and cooling down to room temperature the yellow precipitate was collected by filtration and washed three times with water (20 mL). The product was further washed using methanol (50 mL) and ethylacetate (50 mL) and dried under high vacuum. The purified product was obtained as a yellow solid (0.80 g, 96%). ^{1}H NMR [ppm] (700 MHz, $C_2D_2Cl_4$): 9.12 (d, J^4 = 1.3 Hz, 2H), 8.75 (d, J^3 = 8.3 Hz, 4H), 8.21 (d, J^3 = 8.2 Hz, 4H), 8.06 (t, J^4 = 1.8 Hz, 1H), 7.66 (t, J^3 = 7.2 Hz, 4H), 7.62 (t, J^3 = 7.3 Hz, 2H), 7.56 (t, J^3 = 7.3 Hz, 4H), 7.51 (t, J^3 = 7.3 Hz, 4H), 7.38 (t, J^3 = 7.2 Hz, 4H). ^{13}C NMR [ppm] (176 MHz, $C_2D_2Cl_4$): 172.4, 170.6, 141.0, 140.6, 140.1, 136.0, 132.9, 129.4, 128.9, 128.8, 126.6, 126.3, 123.9, 120.8, 120.5, 109.9. MALDI calc. for $C_{45}H_{29}N_5$ 649.24, found 639.27.

9,9'-(5-(4,6-bis(4-fluorophenyl)-1,3,5-triazin-2-yl)-1,3-phenylene)bis(9*H*-carbazole) (2CzTrz-F)

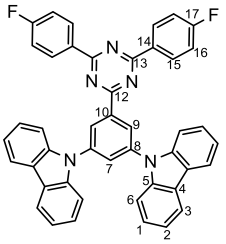

According to the general procedure 9,9'-(5-(4,4,5,5-tetramethyl-1,3,2-dioxaborolan-2-yl)-1,3-phenylene)bis(9*H*-carbazole) (1.51 g, 2.83 mmol), 2-chloro-4,6-bis(4-fluorophenyl)-1,3,5-triazine (0.94 g, 3.11 mmol), K_2CO_3 (1.17 g, 8.49 mmol) and $Pd(PPh_3)_4$ (164 mg, 0.14 mmol) were used and dissolved in THF/water (20:10 mL). After completion of the reaction and

cooling down to room temperature the yellow precipitate was collected by filtration and washed three times with water (40 mL). The product was further washed using methanol (100 mL) and ethylacetate (100 mL) and dried under high vacuum. The purified product was obtained as a yellow solid (1.88 g, 98%). ^{1}H NMR [ppm] (700 MHz, $C_2D_2Cl_4$): 9.07 (H^9, d, J^4 = 1.3 Hz, 2H), 8.75 (H^{15}, d, J^3 = 8.3 Hz, 4H), 8.21 (H^3, d, J^3 = 8.3 Hz, 4H), 8.06 (H^7, t, J^4 = 1.8 Hz, 1H), 7.64 (H^6, t, J^3 = 8.2 Hz, 4H), 7.51 (H^1, t, J^3 = 7.3 Hz, 4H), 7.38 (H^2, t, J^3 = 7.3 Hz, 4H), 7.24 (H^{16}, d, J^3 = 8.2 Hz, 4H). ^{13}C NMR [ppm] (176 MHz, $C_2D_2Cl_4$): 171.6 (C^{13}), 170.6 (C^{12}), 165.7 (C^{17}) , 140.9 (C^5), 140.5 (C^8), 140.2 (C^{10}), 132.1 (C^{14}), 131.6 (C^{15}), 129.2 (C^7), 126.7 (C^1), 126.4 (C^9) , 123.8 (C^4), 120.7 (C^2) , 120.4 (C^3) , 115.9 (C^{16}), 109.7 (C^6). MALDI calc. for $C_{45}H_{27}F_2N_5$ 675.22, found 675.42.

9-(4-(4,6-diphenyl-1,3,5-triazin-2-yl)-2,6-dimethylphenyl)-9*H*-carbazole (1CzTrz)

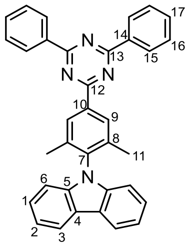

According to the general procedure 9-(2,6-dimethyl-4-(4,4,5,5-tetramethyl-1,3,2-dioxaborolan-2-yl)phenyl)-9*H*-carbazole (0.63 g, 1.59 mmol), 2-chloro-4,6-diphenyl-1,3,5-triazine (0.47 g, 1.75 mmol), K_2CO_3 (0.66 g, 4.78 mmol) and $Pd(PPh_3)_4$ (92 mg, 0.08 mmol) were used and dissolved in THF/water (10:5 mL). The purified product was obtained as a white solid (0.57 g, 71%). ^{1}H NMR [ppm] (700 MHz, $C_2D_2Cl_4$): 8.81 (d, J^3 = 8.5 Hz, 4H), 8.67 (s, 2H), 8.20 (d, J^3 = 8.3 Hz, 2H), 7.67 (t, J^3 = 7.2 Hz, 2H), 7.63 (t, J^3 = 7.4 Hz, 4H), 7.43 (t, J^3 = 7.3 Hz, 2H), 7.31 (t, J^3 = 7.2 Hz, 2H), 7.02 (d, J^3 = 8.2 Hz, 2H) 2.05 (s, 6H). ^{13}C NMR [ppm] (176 MHz, $C_2D_2Cl_4$): 171.8, 171.4, 140.1, 138.8, 138.7, 136.3, 136.1, 133.0, 129.4, 129.2, 128.9, 126.4, 123.1, 120.7, 119.8, 109.7, 18. MALDI calc. for $C_{35}H_{26}N_4$ 502.22, found 502.24.

9-(4-(4,6-bis(4-fluorophenyl)-1,3,5-triazin-2-yl)-2,6-dimethylphenyl)-9*H*-carbazole (1CzTrz-F)

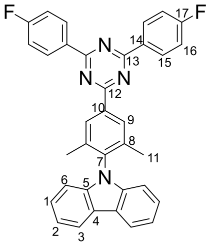

According to the general procedure 9-(2,6-dimethyl-4-(4,4,5,5-tetramethyl-1,3,2-dioxaborolan-2-yl)phenyl)-9*H*-carbazole (0.58 g, 1.46 mmol), 2-chloro-4,6-bis(4-fluorophenyl)-1,3,5-triazine (0.49 g, 1.61 mmol), K_2CO_3 (0.61 g, 4.38 mmol) and $Pd(PPh_3)_4$ (84 mg, 0.07 mmol) were used and dissolved in THF/water (10:5 mL). The purified product was obtained as a white solid (0.55 g, 70%). ^{1}H NMR [ppm] (700 MHz, $C_2D_2Cl_4$): 8.81 (H^{15}, t, J^3_{H-H}=6.8 Hz, 4H), 8.62 (H^9, s, 2H), 8.19 (H^3, d, J^3_{H-H}=7.7 Hz, 2H), 7.42 (H^1 ,t, J^3_{H-H} = 7.8 Hz, 2H), 7.31 (H^{16} , t, J^3_{H-H}=7.1 Hz, 4H), 7.29 (H^2, t, J^3_{H-H}=8.3 Hz, 2H), 6.99 (H^6 ,d, J^3_{H-H}=8.1 Hz, 2H), 2.03 (H^{11}, s, 6H). ^{13}C NMR [ppm] (176 MHz, $C_2D_2Cl_4$): 171.4 (C^{12}, s, 1C), 170.8 (C^{13}, s, 2C), 165.9 (C^{17}, d, J^1_{C-F}=251.7 Hz, 2C), 140.2 (C^5, s, 2C), 138.9 (C^8, s, 2C), 138.8 (C^7, s, 1C), 136.2 (C^{10}, s, 1C), 132.1 (C^{14}, d, J^4_{C-F} = 3 Hz, 2C), 131.7 (C^{15}, d, J^3_{C-F}=8.1 Hz, 4C), 129.1 (C^9, s, 2C), 126.4 (C^1, s, 2C), 123.1 (C^4, s, 2C), 120.7 (C^3, s, 2C), 119.8 (C^2, s, 2C), 115.9 (C^{16}, d, J=21.2 Hz, 4C), 109.5 (C^6, s, 2C), 18.1 (C^{11}, s, 2C). MALDI calc. for $C_{35}H_{24}F_2N_4$ 649.24, found 538.20.

General procedure for the formation of triazine.[56] Benzamidine hydrochloride (2.0 eq), copper acetate (1.0 eq), sodium carbonate (2.0 eq) and the corresponding fluorobenzaldehyde (1.0 eq.) were placed together in an open flask and suspended in toluene. The suspension was heated at 100 °C over night. The reaction mixture was cautiously treated with 2M-HCl solution until all residues were dissolved. The water phase was extracted with DCM for three times. The combined organic phases were washed with NH_4Cl solution, brine solution and dried over $MgSO_4$. The product was purified by column chromatography (stationary phase: silica, eluent: hexane/DCM).

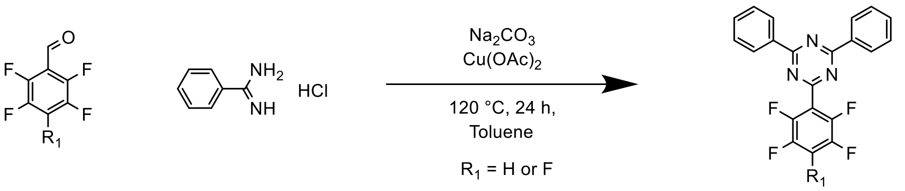

2,4-diphenyl-6-(2,3,5,6-tetrafluorophenyl)-1,3,5-triazine

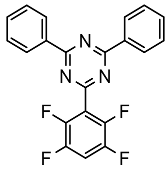

According to the general procedure 2,3,5,6-tetrafluorobenzaldehyde (1.00 g, 5.6 mmol), benzamidine hydrochloride (1.76 g, 11.20 mmol), sodium carbonate (1.19 g, 11.20 mmol) and copper acetate (1.10 g, 5.6 mmol) were suspended in toluene (40 mL). The purified product was obtained as a light grey solid (0.77 g, 36%). ^{1}H NMR (700 MHz, $C_2D_2Cl_4$) [ppm]: 8.68 (d, J^3 = 7.8 Hz, 4H), 7.64 (t, J^3 = 7.1 Hz, 2H), 7.58 (t, J^3 = 7.1 Hz, 4H), 7.30 (tt, $J^3_{H\text{-}F}$ = 6.9, J^4 = 9.1 Hz, 1H). ^{13}C NMR (176 MHz, $C_2D_2Cl_4$) [ppm]: 172.4, 167.1, 146.9, 145.8, 145.5, 144.3, 135.2, 133.5, 129.1, 118.7, 107.9. ^{19}F NMR (658 MHz, $C_2D_2Cl_4$): -137.6, -141.5. APCI calc. for $C_{21}H_{11}F_4N_3$ 381.09, found: 382.20.

2-(perfluorophenyl)-4,6-diphenyl-1,3,5-triazine

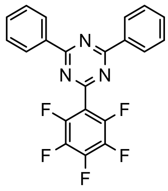

According to the general procedure 2,3,4,5,6-pentafluorobenzaldehyde (1.00 g, 5.07 mmol), benzamidine hydrochloride (1.59 g, 10.15 mmol), sodium carbonate (1.08 g, 10.15 mmol) and copper acetate (1.00 g, 5.07 mmol) were suspended in toluene (40 mL). The purified product was obtained as a light grey solid (0.73 g, 36%). ^{1}H NMR [ppm] (700 MHz, $C_2D_2Cl_4$): 8.67 (d, J^3 = 7.6 Hz, 4H), 7.65 (t, J^3 = 7.3 Hz, 2H), 7.58 (t, J^3 = 7.3 Hz, 4H). ^{13}C NMR [ppm] (176 MHz, $C_2D_2Cl_4$): 171.9, 166.3, 146.5, 145.0, 143.3, 141.7, 138.8, 137.2, 135.1, 133.5, 131.8, 130.0, 113.6. ^{19}F NMR [ppm] (658 MHz, $C_2D_2Cl_4$): -140.5, -150.5, -160.6. APCI calc. for $C_{21}H_{10}F_5N_3$ 399.08, found 400.20.

General procedure for introduction of carbazole in triazine containing fluorobenzenes.

A similar procedure to the previously mentioned general procedure for introduction of carbazole was performed with higher amount of Cs_2CO_3. A mixture of carbazole (5 Cz: 5 eq., 4 Cz: 4 eq.) and Cs_2CO_3 (5 Cz: 20 eq., 4 Cz: 16 eq.) was placed in a two necked flame-dried flask under an argon atmosphere, suspended in DMF and stirred at room temperature for 30 min, followed by addition of corresponding fluorobenzene (5 Cz: 1 eq., 4 Cz: 1 eq.) and heating to 175 °C for 24 h. After cooling to room temperature, the reaction mixture was poured on water and the water phase was extracted three times with dichloromethane (DCM). The combined organic phases were washed with brine solution and dried over $MgSO_4$. The solvent was evaporated under reduced pressure and the crude product was purified by column chromatography (stationary phase: silica, eluent: hexane/DCM 3:2).

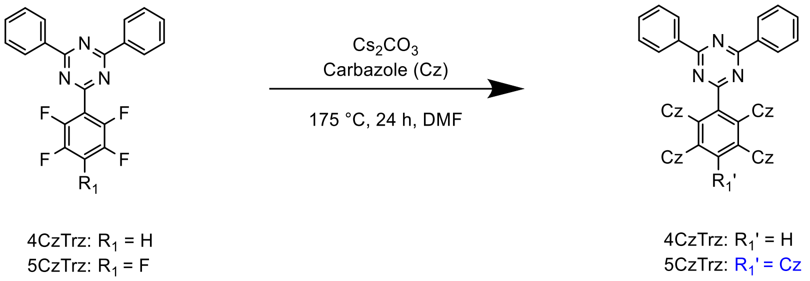

<u>9,9',9'',9'''-(3-(4,6-diphenyl-1,3,5-triazin-2-yl)benzene-1,2,4,5-tetrayl)tetrakis(9*H*-carbazole) (4CzTrz)</u>

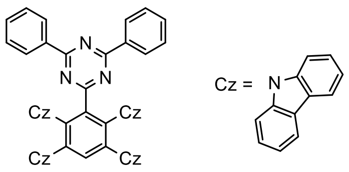

According to the general procedure 2,4-diphenyl-6-(2,3,5,6-tetrafluorophenyl)-1,3,5-triazine (0.20 g, 0.52 mmol), carbazole (0.36 g, 2.1 mmol) and Cs_2CO_3 (2.73 g, 8.4 mmol) were suspended in DMF (10 mL). The purified product was obtained as a yellow solid (0.50 g, 98%). ^{1}H NMR [ppm] (700 MHz, $C_2D_2Cl_4$): 8.38 (s, 1H), 7.85 (d, J^3 = 8.4 Hz, 3H), 7.52-7.44 (m, 6H), 7.35 (d, J^3 = 8.4 Hz, 3H), 7.30 (t, J^3 = 7.3 Hz, 2H), 7.27 (d, J^3 = 8.4 Hz, 3H), 7.17 (t, J^3 = 7.2 Hz, 3H), 7.12 (t, J^3 = 7.3 Hz, 3H), 7.07 (t, J^3 = 7.3 Hz, 3H), 7.02 (t, J^3 = 7.2 Hz, 3H), 6.92 (t, J^3 = 7.2 Hz, 2H). ^{13}C NMR [ppm] (176 MHz, $C_2D_2Cl_4$): 170.7, 170.0, 141.6, 140.7, 140.6, 138.6,

135.8, 134.9, 133.1, 131.8, 128.5, 127.9, 125.9, 125.3, 123.9, 123.8, 120.6, 120.1, 120.0, 119.8, 110.4, 110.1. MALDI calc for $C_{69}H_{43}N_7$ 969.36, found 969.40.

9,9',9'',9''',9''''-(6-(4,6-diphenyl-1,3,5-triazin-2-yl)benzene-1,2,3,4,5-pentayl)pentakis(9*H*-carbazole) (5CzTrz)

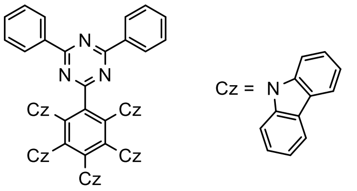

According to the general procedure 2-(perfluorophenyl)-4,6-diphenyl-1,3,5-triazine (0.20 g, 0.52 mmol), carbazole (0.42 g, 2.5 mmol) and Cs_2CO_3 (3.25 g, 10.0 mmol) were suspended in DMF (10 mL). The purified product was obtained as a yellow solid (0.33 g, 55%). ^{1}H NMR [ppm] (700 MHz, $C_2D_2Cl_4$): 7.42 (d, J^3 = 8.0 Hz, 4H), 7.40-7.35 (m, 8H), 7.33-7.10 (m, 12H), 7.05 (t, J^3 = 7.0 Hz, 6H), 6.95 (t, J^3 = 7.0 Hz, 6H), 6.89 (t, J^3 = 7.0 Hz, 6H), 6.84-6.70 (m, 6H), 6.67 (t, J^3 = 7.0 Hz, 2H). ^{13}C NMR [ppm] (176 MHz, $C_2D_2Cl_4$): 170.9, 140.7, 139.3, 138.6, 137.9, 137.7, 134.9, 131.8, 128.4, 127.9, 125.0, 124.5, 124.3, 123.8, 120.1, 120.0, 119.9, 119.7, 119.3, 119.2. MALDI calc for $C_{81}H_{50}N_8$ 1134.42, found 1134.48

2nd series: Synthesis of DTPT-DCz, DTPT-DFCz and DTPT-D2FCz

2,4-di-*tert*-butyl-6-(3,5-difluorophenyl)-1,3,5-triazine

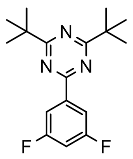

2-chloro-4,6-di-tert-butyl-1,3,5-triazine was prepared according to literature.[57,58] 2-chloro-4,6-di-*tert*-butyl-1,3,5-triazine (1.80 g, 7.9 mmol) was dissolved together with 2-(3,5-difluorophenyl)-4,4,5,5-tetramethyl-1,3,2-dioxaborolane (2.5 g, 10.4 mmol) in anhydrous THF. After stirring for 30 min, $Pd(PPh_3)_4$ (0.41 g, 0.35 mmol) and K_2CO_3 (9.93 g, 71.82 mmol) were added in above mixture. The solution was heated to 85 °C over night. After cooling down to room temperature the reaction mixture was extracted with DCM and washed with water. The solvent was removed under reduced pressure and the crude product was purified by column chromatography (stationary phase: silica, eluent: hexane/DCM). The purified product was

obtained as a white solid (1.93 g, 80%). ^{1}H NMR [ppm] (400 MHz, CD_2Cl_2): 8.17 -7.94 (m, 2H), 6.92 (tt, J = 8.7, 2.5 Hz, 1H), 1.35 (s, 18H). ^{13}C NMR [ppm] (101 MHz, CD_2Cl_2): 186.00, 168.56, 162.62, 140.99, 117.79, 107.42 39.99, 29.09. APCI calc. for $C_{17}H_{22}N_3F_2$: 306.1782; found: 306.1784.

General procedure for the formation of fluorocarbazole.[59,60]

Under an argon atmosphere $Cu(OAc)_2$ (0.2 eq) was dissolved together with benzoic acid (10.0 eq), the phenylboronic acid derivative (10 eq.), 4-fluoroaniline (3 eq.) and K_2CO_3 (10 eq.) in ethyl acetate. The reaction mixture was stirred at 80 °C for 4h. The solvent was removed under reduced pressure and the crude product was purified by column chromatography to give a sticky black liquid. $Pd(OAc)_2$ and acetic acid were added and the reaction mixture was heated to reflux for 30 min. After cooling to room temperature, the reaction mixture was filtered through celite. The reaction mixture was extracted with ethyl acetate and the combined organic phases were washed with saturated Na_2CO_3 solution. Removal of the solvent resulted in a brown residue which was purified by column chromatography (stationary phase: silica eluent, hexane/EA 5:1).

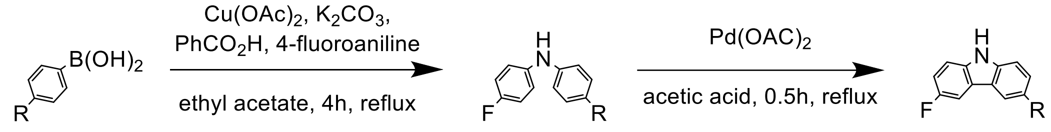

2FCz: R = F
FCz : R = H

<u>**3-fluoro-9*H*-carbazole (FCz)**</u>

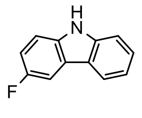

According to the general procedure $Cu(OAc)_2$ (0.363 g, 0.2 mmol), benzoic acid (1.22 g, 10.0 mmol), 4-fluoroaniline (1 mL, 10.0 mmol), phenylboronic acid (3.66 g, 3.0 mmol), and K_2CO_3 (1.38 g, 10.0 mmol) were dissolved in ethyl acetate (50 mL). After reaction and purification the product was obtained as a black liquid. (1.12 g, 6.0 mmol, 60%). Then $Pd(OAc)_2$ (1.35 g, 6.0 mmol) and glacial acetic acid (35 mL) were added. The purified product was obtained as a white solid (45%). ^{1}H NMR [ppm] (400 MHz, CD_2Cl_2): 8.25 – 8.06 (m, 1H),

8.04 (dq, *J* = 7.9, 1.0 Hz, 1H), 7.74 (ddt, *J* = 9.0, 2.5, 0.7 Hz, 1H), 7.50 – 7.37 (m, 3H), 7.29 – 7.14 (m, 2H). ^{13}C NMR [ppm] (101 MHz, CD_2Cl_2): 157.84, 141.03, 136.28, 126.85, 124.14, 123.31, 120.83, 119.79, 113.86, 11.68, 111.33, 106.14. APCI calc. for $C_{12}H_8NF$: 185.0641; found: 185.0640.

3,6-difluoro-9*H*-carbazole (2FCz)

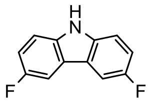

According to the general procedure $Cu(OAc)_2$ (0.363 g, 0.20 mmol), benzoic acid (1.22 g, 10.0 mmol), 4-fluoroaniline (1 mL, 10 mmol) and 4-fluorophenylboronic acid (4.20 g, 3.0 mmol), K_2CO_3 (1.38 g, 10 mmol) in ethyl acetate (50 mL). After reaction and purification the product was obtained as a black liquid (1.29 g, 6.5 mmol, 65%). Then $Pd(OAc)_2$ (1.46 g, 6.5 mmol) and glacial acetic acid (40 mL) were added. The purified product was obtained as a white solid (39%). ^{1}H NMR [ppm] (400 MHz, CD_2Cl_2): 8.09 (s, 1H), 7.59 (ddt, J = 9.0, 2.6, 0.7 Hz, 2H), 7.32 (ddd, J = 8.8, 4.3, 0.5 Hz, 2H), 7.11 (td, J = 9.0, 2.6 Hz, 2H). ^{13}C NMR [ppm] (101 MHz, CD_2Cl_2): 157.69, 137.36, 123.78, 114.66, 112.08, 106.29. APCI calc. for $C_{12}H_7NF_2$:203.0547; found: 203.0547.

General procedure for introduction of carbazole in ditert-butyl-triazine containing fluorobenzenes.

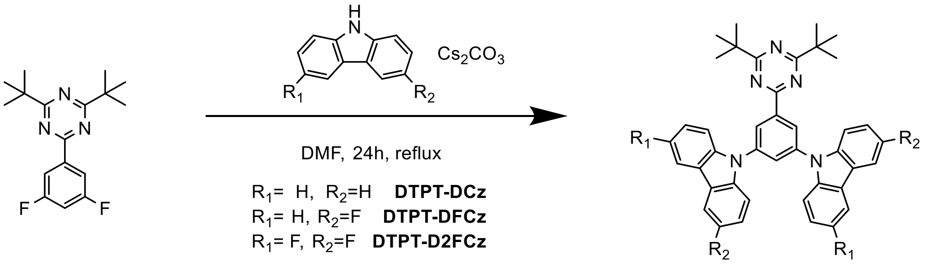

Under argon atmosphere carbazole (2.0 eq.) and Cs_2CO_3 (8.0 eq.) were suspended in DMF (100 mL) at stirred at room temperature for 30 min. 2,4-di-*tert*-butyl-6-(3,5-difluorophenyl)-1,3,5-triazine (1.0 eq.) was added at once and the reaction mixture was heated to reflux for 24 h. After cooling to room temperature the resulting reaction mixture was diluted with water and extracted with chloroform. The combined organic phases were dried over Na_2SO_4 and the

solvent was removed under reduced pressure. The crude product was purified by column chromatography (stationary phase: silica, eluent: hexane/DCM 4:1).

9,9'-(5-(4,6-di-*tert*-butyl-1,3,5-triazine-2-yl)-1,3-phenylene)bis(9*H*-carbazole) (DTPT-DCz)

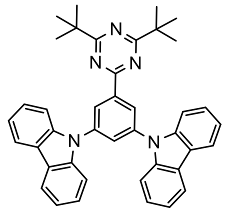

Following the general procedure carbazole (0.334 g, 2.0 mmol) Cs_2CO_3 (2.6 g, 8.0 mmol) and 2,4-di-*tert*-butyl-6-(3,5-difluorophenyl)-1,3,5-triazine (0.305 g, 1.0 mmol) were used in DMF (100 mL). The purified product was obtained as a white solid (64%). ^{1}H NMR [ppm] (300 MHz, $CDCl_3$): 8.93 (d, J = 2.1 Hz, 2H), 8.19 (d, J = 7.7 Hz, 4H), 7.98 (t, J = 2.1 Hz, 1H), 7.59 (d, J = 8.2 Hz, 4H), 7.47 (ddd, J = 8.3, 7.1, 1.3 Hz, 4H), 7.42 – 7.29 (m, 4H), 1.42 (s, 18H). ^{13}C NMR [ppm] (75 MHz, $CDCl_3$): 185.87, 140.73, 139.81, 128.32, 126.43, 126.06, 123.83, 120.66, 120.61, 109.83, 39.87, 29.11. APCI calc. for $C_{41}H_{37}N_5$: 599.3049; found: 599.3042.

9,9'-(5-(4,6-di-*tert*-butyl-1,3,5-triazine-2-yl)-1,3-phenylene)bis(3-fluoro-9*H*-carbazole) (DTPT-DFCz):

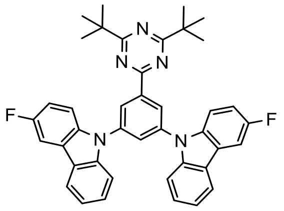

According to the general procedure 3-fluoro-carbazole (0.370 g, 2 mmol), Cs_2CO_3 (2.6 g, 8 mmol) and 2,4-di-tert-butyl-6-(3,5-difluorophenyl)-1,3,5-triazine (0.305 g, 1 mmol) were used in DMF (100 mL). The purified product was obtained as a white solid (58%). ^{1}H NMR [ppm] (300 MHz, $CDCl_3$): 8.90 (d, J = 2.0 Hz, 2H), 8.12 (d, J = 7.8 Hz, 2H), 7.92 (t, J = 2.1 Hz, 1H), 7.82 (dd, J = 8.7, 2.6 Hz, 2H), 7.57 (d, J = 8.2 Hz, 2H), 7.54 – 7.44 (m, 4H), 7.33 (t, J = 7.5 Hz, 2H), 7.19 (td, J = 9.0 Hz, 2.6 Hz, 2H), 1.43 (s, 18H). ^{13}C NMR [ppm] (75 MHz, $CDCl_3$): 185.94, 168.93, 159.81, 156.66, 141.29, 139.76, 137.06, 128.01, 127.06, 126.01, 124.48,

123.39, 120.8, 114.32, 113.99, 110.47, 110.03, 106.43, 39.89, 29.10. APCI calc. for $C_{41}H_{35}N_5F_2$: 635.2861; found: 635.2849.

9,9'-(5-(4,6-di-*tert*-butyl -1,3,5-triazine-2-yl)-1,3-phenylene)bis(3,6-difluoro-9*H*-carbazole (DTPT-D2FCz):

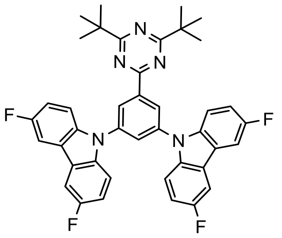

According to the general procedure 3,6-difluoro-carbazole (0.406 g, 2.0 mmol), Cs_2CO_3 (2.6 g, 8.0 mmol) and 2,4-di-tert-butyl-6-(3,5-difluorophenyl)-1,3,5-triazine (0.305 g, 1.0 mmol), were used in DMF (100 mL). The purified product was obtained as a white solid (56%). ^{1}H NMR [ppm] (400 MHz, CD_2Cl_2): 8.90 (d, J = 2.1 Hz, 2H), 7.92 (t, J = 2.1 Hz, 1H), 7.79 (dd, J = 8.7, 2.5 Hz, 4H), 7.52 (dd, J = 9.0, 4.2 Hz, 4H), 7.24 (td, J = 9.0, 2.6 Hz, 4H), 1.43 (s, 18H). ^{13}C NMR [ppm] (101 MHz, CD_2Cl_2): 186.19, 169.14, 158.29, 141.57, 139.89, 138.27, 128.17, 126.37, 124.10, 115.06, 111.21, 106.67, 40.04, 29.05. APCI calc. for $C_{41}H_{33}N_5F_4$: 671.2672; found: 671.2662.

5. References

29. Sun, H. *et al.* Impact of Dielectric Constant on the Singlet-Triplet Gap in Thermally Activated Delayed Fluorescence Materials. *J. Phys. Chem. Lett.* **8**, 2393–2398 (2017).

30. Sun, H. *et al.* Ionization Energies, Electron Affinities, and Polarization Energies of Organic Molecular Crystals: Quantitative Estimations from a Polarizable Continuum Model (PCM)-Tuned Range-Separated Density Functional Approach. *J. Chem. Theory Comput.* **12**, 2906–2916 (2016).

31. Jorgensen, W. L. & Tirado-Rives, J. Potential energy functions for atomic-level simulations of water and organic and biomolecular systems. *Proc. Natl. Acad. Sci. U. S. A.* **102**, 6665–6670 (2005).

32. Jorgensen, W. L., Maxwell, D. S. & Tirado-Rives, J. Development and testing of the OPLS all-atom force field on conformational energetics and properties of organic liquids. *J. Am. Chem. Soc.* **118**, 11225–11236 (1996).

33. Mondal, A. *et al.* Molecular library of OLED host materials—Evaluating the multiscale simulation workflow. *Chem. Phys. Rev.* **2**, 031304 (2021).

34. Cole, D. J., Vilseck, J. Z., Tirado-Rives, J., Payne, M. C. & Jorgensen, W. L. Biomolecular Force Field Parameterization via Atoms-in-Molecule Electron Density Partitioning. *J. Chem. Theory Comput.* **12**, 2312–2323 (2016).

35. Manz, T. A. & Limas, N. G. Introducing DDEC6 atomic population analysis: Part 1. Charge partitioning theory and methodology. *RSC Adv.* **6**, 47771–47801 (2016).

36. Tkatchenko, A. & Scheffler, M. Accurate molecular van der Waals interactions from ground-state electron density and free-atom reference data. *Phys. Rev. Lett.* **102**, 6–9 (2009).

37. Frisch, M. J. *et al.* G16_C01. Gaussian 16, Revision C.01, Gaussian, Inc., Wallin at (2016).

38. Shen, L. Y. *et al.* Improving the Efficiency of Blue Organic Light-Emitting Diodes by Employing Cs-Derivatives as the n-Dopant. *Acta Phys. -Chim. Sin* **28**, 1497–1501 (2012).

39. Neese, F., Wennmohs, F., Becker, U. & Riplinger, C. The ORCA quantum chemistry program package. *J. Chem. Phys.* **152**, (2020).

40. Poelking, C. *et al.* Characterization of charge-carrier transport in semicrystalline polymers: Electronic couplings, site energies, and charge-carrier dynamics in poly(bithiophene-alt-thienothiophene) [PBTTT]. *J. Phys. Chem. C* **117**, 1633–1640 (2013).

41. Abraham, M. J. *et al.* Gromacs: High performance molecular simulations through multi-level parallelism from laptops to supercomputers. *SoftwareX* **1**–**2**, 19–25 (2015).

42. Pronk, S. *et al.* GROMACS 4.5: A high-throughput and highly parallel open source

molecular simulation toolkit. *Bioinformatics* **29**, 845–854 (2013).

43. Bussi, G., Donadio, D. & Parrinello, M. Canonical sampling through velocity rescaling. *J. Chem. Phys.* **126**, (2007).

44. Martinez, L., Andrade, R., Birgin, E. G. & Martínez, J. M. PACKMOL: A package for building initial configurations for molecular dynamics simulations. *J. Comput. Chem.* **30**, 2157–2164 (2009).

45. Poelking, C. & Andrienko, D. Long-Range Embedding of Molecular Ions and Excitations in a Polarizable Molecular Environment. *J. Chem. Theory Comput.* **12**, 4516–4523 (2016).

46. Kordt, P. & Andrienko, D. Modeling of Spatially Correlated Energetic Disorder in Organic Semiconductors. *J. Chem. Theory Comput.* **12**, 36–40 (2016).

47. Rühle, V. *et al.* Microscopic simulations of charge transport in disordered organic semiconductors. *J. Chem. Theory Comput.* **7**, 3335–3345 (2011).

48. Baumeier, B., Kirkpatrick, J. & Andrienko, D. Density-functional based determination of intermolecular charge transfer properties for large-scale morphologies. *Phys. Chem. Chem. Phys.* **12**, 11103–11113 (2010).

49. Rühle, V., Junghans, C., Lukyanov, A., Kremer, K. & Andrienko, D. Versatile Object-Oriented Toolkit for Coarse-Graining Applications. 3211–3223 (2009).

50. Lukyanov, A. & Andrienko, D. Extracting nondispersive charge carrier mobilities of organic semiconductors from simulations of small systems. *Phys. Rev. B - Condens. Matter Mater. Phys.* **82**, 2–5 (2010).

51. Kirkpatrick, J. An Approximate Method for CalculatingTransfer Integrals Based on the ZINDO Hamiltonian. *Int. J. Quantum Chem.* **108**, 51–56 (2007).

52. Andrienko, D. VOTCA-CTP Charge Transport Manual. *www.votca.org* (2019).

53. Oh, C. S., Lee, H. L., Han, S. H. & Lee, J. Y. Rational Molecular Design Overcoming the Long Delayed Fluorescence Lifetime and Serious Efficiency Roll-Off in Blue Thermally Activated Delayed Fluorescent Devices. *Chem. - A Eur. J.* **25**, 642–648

(2019).

54. Cho, Y. J., Yook, K. S. & Lee, J. Y. A universal host material for high external quantum efficiency close to 25% and long lifetime in green fluorescent and phosphorescent OLEDs. *Adv. Mater.* **26**, 4050–4055 (2014).

55. Kim, M., Jeon, S. K., Hwang, S. H. & Lee, J. Y. Stable blue thermally activated delayed fluorescent organic light-emitting diodes with three times longer lifetime than phosphorescent organic light-emitting diodes. *Adv. Mater.* **27**, 2515–2520 (2015).

56. Wang, X. *et al.* Synthesis, Spectra, and Theoretical Investigations of 1,3,5-Triazines Compounds as Ultraviolet Rays Absorber Based on Time-Dependent Density Functional Calculations and three-Dimensional Quantitative Structure-Property Relationship. *J. Fluoresc.* **28**, 707–723 (2018).

57. Hintermann, L., Xiao, L. & Labonne, A. A general and selective copper-catalyzed cross-coupling of tertiary grignard reagents with azacyclic electrophiles. *Angew. Chemie - Int. Ed.* **47**, 8246–8250 (2008).

58. Hintermann, L. *et al.* The AZARYPHOS family of ligands for ambifunctional catalysis: Syntheses and use in ruthenium-catalyzed anti-markovnikov hydration of terminal alkynes. *Chem. - A Eur. J.* **15**, 7167–7179 (2009).

59. Bedford, R. B., Betham, M., Charmant, J. P. H. & Weeks, A. L. Intramolecular direct arylation in the synthesis of fluorinated carbazoles. *Tetrahedron* **64**, 6038–6050 (2008).

60. Woon, K. L., Nadiah, Z. N., Hasan, Z. A., Ariffin, A. & Chen, S. A. Tuning the singlet-triplet energy splitting by fluorination at 3,6 positions of the 1,4-biscarbazoylbenzene. *Dye. Pigment.* **132**, 1–6 (2016).
